# The Use of Psychedelics in the Treatment of Adult ADHD: A Systematic and Mechanistic Review

**DOI:** 10.3390/ijms27083453

**Published:** 2026-04-12

**Authors:** James Chmiel, Agnieszka Malinowska, Donata Kurpas

**Affiliations:** 1 Institute of Physical Culture Sciences, Faculty of Physical Culture and Health, University of Szczecin, Al. Piastów 40B Block 6, 71-065 Szczecin, Poland; 2 Institute of Psychology, University of Szczecin, 71-017 Szczecin, Poland; 3 Division of Research Methodology, Department of Nursing, Faculty of Nursing and Midwifery, Wroclaw Medical University, 51-618 Wrocław, Poland

**Keywords:** psychedelics, ADHD, pharmacotherapy, neurodevelopmental disorders

## Abstract

Interest in classical psychedelics as potential treatments for ADHD has grown alongside broader psychiatric psychedelic research, but ADHD-specific evidence remains limited. This systematic review examined prospective and experimental studies on whether classical psychedelics, including microdosing-like use and retreat-based exposure, are associated with changes in adult ADHD symptoms and related functioning. A PRISMA-guided systematic review was conducted using a PECO/PICO framework focused on adults (≥18 years) with diagnosed ADHD and/or elevated ADHD symptomatology who were exposed to a classical psychedelic and assessed prospectively with quantitative ADHD outcomes. Major databases were searched, with reference screening and targeted checks for recent or registered trials. Risk of bias was assessed using RoB 2 for the RCT and ROBINS-I for non-randomized studies. Because of heterogeneity and the small number of studies, findings were synthesized narratively. Five studies met the inclusion criteria. Five prospective/experimental studies were included: three naturalistic online microdosing cohorts, one randomized double-blind placebo-controlled phase 2A trial of low-dose LSD, and one pre-post ayahuasca retreat pilot. In uncontrolled naturalistic microdosing studies, participants reported short-term reductions in ADHD symptom ratings together with improvements in well-being and affect-related functioning; however, these studies were highly vulnerable to self-selection, expectancy, attrition, and non-standardized exposure. In contrast, the only randomized placebo-controlled ADHD trial found improvement in both LSD and placebo groups, with no statistically significant advantage for LSD on clinician-rated or self-reported ADHD outcomes. Objective cognitive findings were limited and inconsistent, and safety data outside the supervised trial context were sparse. Naturalistic studies provide, at most, low-certainty signals of perceived short-term improvement, but the strongest controlled evidence does not demonstrate drug-specific efficacy of repeated low-dose LSD for core ADHD symptoms. Current evidence therefore does not allow separation of pharmacological effects from expectancy, setting, self-monitoring, and broader experiential/contextual influences, and is insufficient to support psychedelics as an evidence-based treatment for ADHD.

## 1. Introduction

Attention-deficit/hyperactivity disorder (ADHD) is a neurodevelopmental condition marked by developmentally inappropriate levels of inattention and/or hyperactivity–impulsivity that cause clinically significant impairment across settings. Beyond its behavioral presentation, ADHD is widely conceptualized as a disorder of self-regulation that emerges from alterations in brain systems supporting executive control, motivation, and the efficient allocation of attention over time [1,2]. The clinical phenotype is heterogeneous, and symptom expression can shift across development, motivating neurobiological models that emphasize distributed circuitry rather than a single locus of dysfunction [2,3].

A central theme in ADHD neurobiology is dysregulation of catecholamine signaling—particularly dopamine and norepinephrine—within prefrontal and fronto-striatal circuits that support working memory, inhibitory control, and goal-directed behavior [1,4]. These neuromodulatory systems influence the “gain” or signal-to-noise of prefrontal networks, shaping the stability of task goals and the ability to resist distraction; when suboptimal, individuals may show context-dependent lapses in sustained attention and control [1,2]. Complementary accounts highlight alterations in reward processing and reinforcement learning, including heightened sensitivity to delayed reinforcement, which may contribute to impulsive decision-making and difficulties persisting with low-immediacy tasks [5].

More recent systems-level perspectives frame ADHD as involving atypical dynamics among large-scale brain networks, including the default mode network (DMN) and task-positive control networks. Meta-analytic work has linked ADHD to altered resting-state connectivity patterns—often described as reduced segregation or abnormal coupling between the DMN and networks that support executive control and attention [6,7]. While findings vary across methods and samples, extensive reviews emphasize that ADHD-related differences may be better understood as network-level coordination problems rather than as uniform “under-” or “over-” activation of a single region [6,8]. These circuit and network models are relevant to treatment development because they suggest multiple potential targets—catecholaminergic modulation, reward/motivation processes, and network organization—that may map onto different symptom profiles and functional outcomes [2,4].

Evidence increasingly suggests that ADHD involves not only catecholaminergic (dopamine/noradrenaline) dysregulation, but also clinically relevant alterations in serotonergic neurotransmission [9]. Across biochemical, genetic, and pharmacological lines of research, reviews have highlighted patterns consistent with disrupted serotonin availability and signaling in at least a subset of people with ADHD, alongside meaningful functional interactions between serotonergic and catecholaminergic systems that can shape attention, impulsivity, and emotional regulation [9,10]. Genetic evidence also supports serotonergic involvement: meta-analytic work has examined associations between ADHD and variants in serotonin receptor genes (e.g., 5-HTR1B/5-HTR2A/5-HTR2C), and broader syntheses have discussed serotonergic contributions to ADHD-linked psychiatric and somatic comorbidity profiles [11]. Finally, targeted reviews of the serotonin transporter (5-HTT/SLC6A4) literature have argued that altered transporter function and synaptic serotonin dynamics may be relevant to ADHD pathophysiology, even if findings are heterogeneous across samples and methods [9,12].

Psychedelics are a class of psychoactive compounds best known for producing marked alterations in perception, emotion, cognition, and self-experience. In contemporary biomedical literature, “classic” or serotonergic psychedelics typically refer to compounds such as psilocybin/psilocin, lysergic acid diethylamide (LSD), mescaline, and N, N-dimethyltryptamine (DMT), which share a characteristic pharmacological emphasis on serotonergic receptor signaling [13,14]. Importantly, substances sometimes discussed in adjacent therapeutic contexts (e.g., MDMA or ketamine) have distinct mechanisms and are not “classic” psychedelics in the narrow pharmacological sense, a definitional issue that can materially affect how evidence is grouped and interpreted in a systematic review [14,15].

At the receptor level, classic psychedelics primarily act as agonists or partial agonists at the serotonin 5-HT2A receptor, with downstream effects on cortical excitability and glutamatergic signaling that are believed to contribute to their acute subjective and cognitive effects [14,16]. A growing mechanistic literature also links psychedelic exposure to markers of neuroplasticity in preclinical and translational work, supporting hypotheses that these compounds may transiently increase the capacity for learning and revision of entrenched patterns [14,17]. While the field continues to refine causal pathways, converging models describe psychedelics as perturbing hierarchical brain processing in ways that can alter attention, meaning-making, and emotional learning—domains that overlap conceptually with core ADHD impairments [13,18].

At the systems neuroscience level, psychedelics have been associated with changes in functional connectivity and network organization, including modulation of the DMN and altered integration among typically segregated networks [18,19]. Reviews synthesize evidence that psilocybin can reduce within-network DMN connectivity acutely while altering broader network interactions. These effects have been discussed as potential neural correlates of changes in self-referential processing and cognitive flexibility [18,19]. In clinical research, psychedelics are most often studied within structured therapeutic frameworks that include preparation, supervised dosing sessions, and integration, reflecting the widely recognized importance of “set and setting” (i.e., mindset and context) in shaping both safety and outcomes [20,21]. This context-sensitivity has direct implications for evaluating the ADHD literature: outcomes may depend not only on pharmacology, but also on psychotherapy models, participant characteristics, comorbidities, and how attentional and functional endpoints are operationalized [20,22].

Microdosing refers to the repeated, intermittent administration of very low doses of a psychedelic—most commonly LSD or psilocybin—usually in amounts intended to produce minimal or no marked acute perceptual alteration, often roughly around 5–20% of a typical full psychoactive dose [23]. However, there is no universally accepted definition, and actual practice varies substantially across studies and community use [23]. Research interest in microdosing has grown because it is hypothesized to influence mood, attention, and cognitive-emotional functioning without the intense subjective effects associated with higher-dose psychedelic sessions [24]. More broadly, the literature on microdosing in psychopathology remains methodologically constrained and clinically inconclusive [25], with naturalistic studies often reporting more favorable outcomes than controlled studies and with persistent concerns regarding expectancy effects, blind breaking, and heterogeneous dosing [26]. However, the current review emphasizes that microdosing should not be treated as a simple low-intensity version of full-dose psychedelic treatment: dosing schedules, subjective effects, expectancy, and contextual factors differ, and controlled studies have often found smaller and less consistent effects than naturalistic reports [27]. Accordingly, mechanisms proposed for high-dose psychedelic interventions—such as profound acute experiential effects, altered self-processing, or psychotherapy-assisted meaning-making—may not translate directly to repeated low-dose regimens, and microdosing must be considered a distinct therapeutic model in its own right [28,29,30,31]. Based on controlled-study reviews, the evidence for microdosing efficacy in psychiatric conditions remains extremely limited, because most placebo-controlled studies to date have been conducted in non-clinical populations, are small, and primarily assess acute or short-term outcomes rather than durable clinical change. A 2024 review of placebo-controlled low-dose LSD and psilocybin studies identified only 19 controlled studies and concluded that it is not yet possible to determine whether microdosing effects are pharmacologically specific or placebo-related; importantly, the same review noted that controlled research has so far provided mixed evidence, with almost no data on sustained clinical effects and no clinical-population evidence at that time [29,31]. Controlled experimental work also does not provide compelling support for improvement in executive or cognitive functioning. In a double-blind placebo-controlled psilocybin microdosing study, low doses produced noticeable subjective effects but no evidence of enhanced well-being, creativity, or cognitive function, with some small changes trending toward impairment; the authors concluded that expectancy likely explains at least part of the anecdotal benefit [32]. Likewise, the large self-blinding citizen-science study found little difference between placebo and microdose conditions on psychological and cognitive outcomes, despite marked improvement from baseline across groups [30].

Most ADHD-related research on psychedelics has not examined high-dose psychedelic treatment models, but rather repeated low-dose or microdosing-like use, often in naturalistic settings. This distinction is important because mechanisms commonly proposed in the broader psychedelic literature—such as profound acute subjective effects, marked alterations in self-processing, or psychotherapy-assisted psychological change—may not translate directly to low-dose regimens and, to date, have not been established for microdosing in ADHD. Accordingly, the rationale for the present review is not that psychedelics have a demonstrated mechanism of benefit in ADHD, but that growing clinical and public interest has outpaced critical synthesis of the available evidence. A focused review is therefore needed to clarify what has been studied in ADHD, which dosing models have been used, what outcomes have been reported, and how strongly study design, expectancy effects, and heterogeneity limit those findings.

This systematic review aims to synthesize and critically evaluate the available evidence on the efficacy of psychedelics in the treatment of ADHD. We examine the included studies in terms of participant characteristics, the psychedelic compounds and dosing approaches used, and their effects across multiple ADHD-related outcomes. The findings are discussed within the broader context of the emerging use of psychedelics in psychiatry. In Section 5, we further explore plausible mechanisms through which psychedelics may influence ADHD symptomatology and associated functional domains. Importantly, the mechanistic discussion in this review should not be read as evidence of established therapeutic action in ADHD. ADHD is not primarily conceptualized as a serotonergic disorder. Although serotonergic systems interact with catecholaminergic and network-level processes relevant to attention and self-regulation, any ADHD-specific therapeutic implications of psychedelic pharmacology remain inferential at present. Because the available clinical evidence is dominated by naturalistic, self-selected, self-report studies with heterogeneous exposure conditions, the mechanistic rationale should be understood as hypothesis-generating rather than confirmatory. Accordingly, throughout this review, we interpret neurobiological plausibility cautiously and give greatest weight to disorder-specific controlled clinical data.

## 2. Materials and Methods

### 2.1. Study Design and Reporting Framework

This work was conducted as a systematic review of experimental and prospective evidence examining the effects of classical psychedelics on ADHD symptoms and ADHD-relevant functioning in adults. Given the small and methodologically heterogeneous evidence base (controlled clinical trial data, naturalistic prospective cohorts, and pre–post designs), the review followed a structured systematic approach to the identification, selection, and appraisal of studies. It used a narrative synthesis, with primarily structured tabulation of study characteristics and outcomes. Reporting was guided by the PRISMA (Preferred Reporting Items for Systematic Reviews and Meta-Analyses) principles, including explicit eligibility criteria, transparent screening procedures, and documented reasons for exclusion at the full-text stage.

### 2.2. Review Question and Analytic Framework (PICO/PECO)

Because included studies operationalized “psychedelic intervention” in different ways (self-directed microdosing, supervised low-dose dosing, and retreat-based ceremonial use), the review used a broad exposure framework.

To reduce outcome-selection bias, the outcome framework was defined a priori from the review question, before study selection and data extraction. The review was designed to capture not only core ADHD symptom severity, but also prespecified domains considered clinically relevant to ADHD and plausibly targeted in psychedelic/microdosing studies, namely emotional and psychosocial functioning, selected cognitive performance outcomes relevant to ADHD, and safety/tolerability. These domains were chosen on conceptual grounds based on the ADHD literature and the broader psychedelic literature, in which studies frequently assess symptom severity together with well-being, emotion regulation, self-related functioning, and adverse effects. Therefore, the outcome categories used in this review were not created post hoc from the included studies, although the specific instruments available within each category necessarily depended on what individual studies reported.

- Population (P): Adults (≥18 years) with clinically diagnosed ADHD, and/or adults with clinically elevated ADHD symptomatology (e.g., screening-scale elevations) when the study explicitly targeted ADHD-like symptoms or ADHD self-management.
- Exposure/Intervention (I/E): Administration or use of a classical psychedelic (e.g., LSD, psilocybin/psilocin-containing preparations, DMT-containing ayahuasca), including microdosing-like regimens and retreat-based ceremonial consumption.
- Comparators (C): Placebo or control conditions where available (e.g., placebo-controlled trial), treatment-as-usual pharmacotherapy comparison groups, or within-person baseline in pre–post designs.
- Outcomes (O): Primary outcomes were ADHD symptom severity (self-report and/or clinician-rated). Secondary outcomes were prespecified as emotional and psychosocial functioning (well-being, emotion regulation, empathy, mindfulness, personality), cognitive performance tasks reported (e.g., timing/time perception), and safety/tolerability indicators (adverse events, discontinuation, screening exclusions, or other risk-management features).

### 2.3. Eligibility Criteria

#### 2.3.1. Inclusion Criteria

Studies were eligible if they met all of the following:

1. Population: Adult participants, either (a) clinically diagnosed with ADHD, or (b) recruited because of elevated ADHD symptoms/impairment and assessed using a validated ADHD symptom instrument.
2. Exposure: A classical psychedelic exposure was clearly present and prospectively related to outcome measurement (e.g., supervised dosing regimen; self-initiated microdosing followed longitudinally; retreat participation including ayahuasca ceremonies).
3. Design: Experimental, quasi-experimental, or prospective observational designs with at least one pre-specified follow-up assessment after exposure initiation (including randomized controlled trials, non-randomized comparative designs, and prospective pre–post designs).
4. Outcomes: Studies had to report at least one quantitative, prospectively assessed ADHD-related outcome using a validated instrument. For this review, the primary eligible outcome was a change in ADHD symptom severity. In addition, based on the prespecified analytic framework, studies were eligible if they reported secondary ADHD-relevant domains such as emotional/psychosocial functioning, cognitive performance relevant to ADHD, or safety/tolerability outcomes, provided that ADHD symptomatology remained a target construct of the study. Examples of eligible ADHD symptom instruments included the Conners’ Adult ADHD Rating Scale (CAARS) variants, the Adult ADHD Self-Report Scale (ASRS), and the Adult Investigator Symptom Rating Scale (AISRS). Instrument names are provided as examples of eligible validated measures and not as an exhaustive list.

#### 2.3.2. Exclusion Criteria

Studies were excluded if they met any of the following:

- Participants were exclusively minors (<18 years).
- The substance was not a classical psychedelic (e.g., studies focused only on MDMA or ketamine without a classical psychedelic arm).
- The design was purely retrospective/cross-sectional with no prospective follow-up (e.g., single-time-point surveys of past psychedelic use).
- The report was a narrative opinion piece, editorial, or commentary without original data.
- ADHD was not assessed using a validated instrument and was not a target construct of the study (e.g., general mental health outcomes without ADHD-related measures).

### 2.4. Information Sources and Search Strategy

A comprehensive literature search was conducted across major medical, psychological, and pharmacological databases (e.g., PubMed/MEDLINE, Embase, PsycINFO, and Web of Science). To reduce publication and indexing bias in an emerging field, additional sources were considered, including reference-list screening of included articles and relevant reviews. They targeted searches for registered or recently completed trials where applicable.

Search strings combined controlled vocabulary (where available) and free-text terms for both the exposure and condition domains. For ADHD, we used broad disorder terms (e.g., “ADHD”, “attention-deficit/hyperactivity disorder”, “inattention”, “hyperactivity”, “impulsivity”). Also, we included the names of commonly used adult ADHD instruments: Conners/CAARS (Conners’ Adult ADHD Rating Scale), ASRS (Adult ADHD Self-Report Scale), and AISRS (Adult Investigator Symptom Rating Scale). These instrument terms were included a priori because potentially relevant studies in this area may be indexed, described in titles/abstracts, or reported primarily by scale name rather than by the full disorder term alone. The use of instrument names was intended to improve the sensitivity of the search in a small and emerging literature, not to restrict eligibility to particular scales.

Search strings combined controlled vocabulary (where available) and free-text terms spanning:

psychedelics: psychedelic, LSD, lysergic acid diethylamide, psilocybin, psilocin, ayahuasca, dimethyltryptamine, DMT, hallucinogen, microdose, microdosing, low dose, sub-perceptual.

ADHD: ADHD, attention-deficit/hyperactivity disorder, attention deficit hyperactivity disorder, inattention, hyperactivity, impulsivity, Conners, CAARS, ASRS, AISRS.

Search terms were adapted to each database’s syntax. No assumptions were made that “microdosing” studies would always use the term explicitly; therefore, combinations of substance terms with “low dose,” “sub-perceptual,” and related descriptors were also included. Records were managed in a reference software system, and duplicates were removed before screening.

### 2.5. Study Selection Process

Study selection proceeded in two stages:

1. Title/abstract screening: Two-step relevance screening was conducted against inclusion criteria, prioritizing sensitivity (erring toward inclusion when eligibility was unclear). The screening process was performed by two reviewers.
2. Full-text review: Potentially eligible articles were assessed. Reasons for exclusion at this stage were recorded (e.g., wrong population, wrong design, no ADHD outcomes, not a classical psychedelic exposure, retrospective-only reporting). The screening process was performed by two reviewers.

When multiple reports appeared to draw from the same recruitment stream or baseline cohort (as was the case for the prospective online microdosing studies described in the evidence base), reports were linked and treated as related publications, with care taken to avoid double-counting participants while still extracting distinct outcomes reported across papers.

### 2.6. Data Extraction and Management

A standardized extraction framework was used to collect:

- Study characteristics: country/setting (e.g., remote online cohort, outpatient clinical sites, retreat location), design (RCT vs. naturalistic), follow-up duration, and recruitment period, where reported.
- Participant characteristics: baseline and follow-up sample sizes, age, sex/gender distribution, diagnostic status (formal diagnosis vs. elevated symptoms), comorbidities, medication status (including washout or concurrent ADHD pharmacotherapy), and prior psychedelic exposure, where available.
- Exposure/intervention details: substance(s) used, dosing model (self-directed microdosing vs. fixed-dose supervised regimen vs. retreat ceremonies), dose and frequency where reported, supervision/monitoring, and key contextual features (e.g., abstinence requirements at retreat, trial safety procedures).
- Outcomes: instruments used, assessment time points, and quantitative results sufficient to characterize change (means/SDs, change estimates, mixed-model effects, or comparable statistics). Outcomes were grouped into:
  1. ADHD symptom severity
  2. Emotional/psychosocial functioning
  3. Cognitive performance/time perception
  4. Safety/tolerability and acceptability
- Methodological features: comparator characteristics (placebo, TAU, or within-person), attrition, handling of missing data, and whether expectancy/blinding integrity was assessed (especially relevant for microdosing-like protocols).

Extraction was performed at the level of the analytic sample used in each report (e.g., “microdosing-only” subgroup vs. combined-use subgroup) to preserve the internal validity of the comparisons described in each study. The extraction categories followed the prespecified outcome framework described in Section 2.2 and were applied consistently across all potentially eligible studies, rather than being derived from the final included reports.

### 2.7. Outcomes and Effect Handling

#### 2.7.1. Primary Outcome

The primary outcome was the change in ADHD symptom severity from baseline to follow-up and/or between-group differences at comparable time points. Because the included evidence used different instruments and rating sources (self-report screening scales versus clinician-rated severity), results were extracted and interpreted within the context of each instrument rather than forcing direct numeric equivalence across scales.

#### 2.7.2. Secondary Outcomes

Secondary outcomes were prespecified before data extraction as ADHD-relevant domains beyond core symptom severity. These included the following: (1) emotional and psychosocial functioning, such as well-being, emotion regulation, empathy-related constructs, mindfulness, and personality traits; (2) cognitive performance outcomes relevant to ADHD, where assessed; and (3) safety/tolerability outcomes, including adverse events, discontinuations, physiological monitoring, screening exclusions, and other reported risk-management features. These categories were defined a priori at the domain level; the specific measures included in each category were study-dependent and were extracted only when reported.

#### 2.7.3. Effect Metrics and Comparability

Where studies reported inferential models (e.g., mixed-effects models for repeated measures), model-reported effects were extracted (time effects; group × time interactions). Where only pre–post summary statistics were available, direction and magnitude of change were summarized. Formal meta-analysis was not prioritized due to the small number of studies and substantial heterogeneity across intervention models, populations (clinically diagnosed ADHD vs. predominantly non-diagnosed retreat attendees), outcome instruments, and comparator structures.

### 2.8. Risk of Bias Assessment

Risk of bias was evaluated using design-appropriate tools:

- Randomized controlled trial: Cochrane Risk of Bias 2 (RoB 2), covering randomization process, deviations from intended interventions (including blinding integrity challenges), missing outcome data, outcome measurement, and selective reporting.
- Non-randomized comparative study (e.g., microdosing vs. TAU): ROBINS-I (Risk Of Bias In Non-randomized Studies of Interventions), with particular emphasis on confounding (baseline group differences such as medication status, sex distribution, and prior psychedelic exposure), selection bias, and outcome measurement.

Given the known expectancy sensitivity and masking challenges in psychedelic studies—especially for low-dose regimens—special attention was paid to risks arising from unblinding, expectancy effects, and self-selection into exposure, alongside attrition in fully remote cohorts.

### 2.9. Data Synthesis Strategy

A structured narrative synthesis was conducted, organized by outcome domain and study type:

1. Population and setting: clinically diagnosed ADHD trial participants vs. naturalistic community cohorts vs. retreat attendees.
2. Intervention model: self-directed microdosing; supervised fixed-dose low-dose LSD; ceremonial retreat ayahuasca exposure.
3. Outcome domain: ADHD symptoms, psychosocial functioning, cognition, safety/tolerability.

To aid interpretability, emphasis was placed on:

- patterns consistent across multiple naturalistic studies (e.g., short-term self-reported symptom reductions during microdosing initiation),
- contrasts between uncontrolled signals and controlled evidence (e.g., placebo-controlled trial outcomes), and
- methodological factors likely to explain discrepancies (expectancy, selection, measurement reactivity, attrition, and non-equivalence of populations).

Where a study included a comparator group (placebo or TAU), those comparisons were deemed to have higher informational value than within-person changes alone. However, causal language was avoided for observational designs.

### 2.10. Systematic Review Registration

The review was registered in the PROSPERO database (CRD420261302347).

## 3. Results

**Figure 1:**
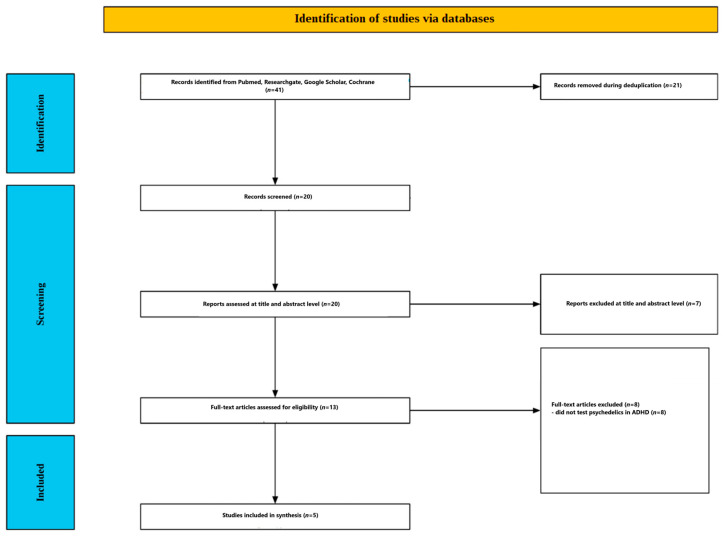
Flow chart depicting the different phases of the systematic review.

Figure 1 summarizes the screening process. The initial search identified 41 records. After removing duplicates, 20 unique records were screened by title and abstract. Seven records were excluded at this stage because they did not examine the use of psychedelics in the treatment of ADHD. Thirteen full-text articles were assessed for eligibility. Eight articles were excluded because they did not investigate psychedelics in ADHD. Ultimately, five studies were included in the review [33,34,35,36,37]. The included studies are presented in Table 1 and Table 2.

### 3.1. Participants’ Characteristics

Across the included evidence base, participant populations ranged from clinically diagnosed adults with moderate-to-severe ADHD enrolled in a controlled outpatient trial to broader community samples with elevated ADHD-like symptomatology participating in naturalistic microdosing or retreat contexts. The three prospective online microdosing studies derived from the same recruitment stream and baseline cohort recruited via a microdosing information website between November 2020 and July 2021 (Maastricht University ethics approval). They therefore shared highly similar baseline characteristics and eligibility thresholds [33,34,35]. In these naturalistic cohorts, adults were eligible if they reported a formal ADHD diagnosis or, if not diagnosed, reported symptom levels severe enough to interfere with daily functioning and met a clinical elevation threshold on the Conners’ Adult ADHD Rating Scale short screening version (CAARS-S:SV; T-score ≥ 65 on at least one subscale) [33,34,35]. After exclusions for non-serious responding and subthreshold symptoms in non-diagnosed respondents, the analyzed baseline cohort comprised 233 participants, with substantial attrition at follow-up typical of fully remote longitudinal surveys (two-week follow-ups in the mid-60s and four-week follow-ups in the mid-40s) [33,34,35]. Baseline participants were in their mid-30s on average (mean ~35 years), with a roughly balanced sex/gender distribution (approximately half female and just under half male, with a small minority identifying as “other”) and a predominantly European residence (a little over four in five) [33,34,35]. Educational attainment was high, with most reporting tertiary education, and occupational backgrounds were diverse, spanning office/computer-based work, study, people-oriented work, creative roles, physical work, and other categories [33,35]. Clinically, these samples were enriched for ADHD but heterogeneous in comorbidity: ADHD was the most frequently endorsed diagnosis (approximately two thirds of the analyzed baseline sample), and a large majority reported at least one current psychiatric, neurological, or physical diagnosis overall, with more than half of ADHD-diagnosed participants reporting at least one additional comorbid condition (commonly depression, anxiety disorders, PTSD, and dyslexia) [33,34,35]. Conventional ADHD medication histories were mixed; among those with an ADHD diagnosis, many reported prior stimulant treatment that had been discontinued, about one third reported current prescribed medication use at baseline, and a smaller subset reported never having used ADHD medication, with amphetamine- and methylphenidate-based preparations most common when medication was used [33,34,35]. Prior psychedelic exposure was prevalent in these microdosing cohorts (about four-fifths reporting lifetime psychedelic use), and the observational “microdosing” exposure was self-directed rather than investigator-assigned [33,34,35]. One study package also included a “treatment as usual” (TAU) comparison group to contextualize symptom trajectories during microdosing relative to continued conventional pharmacotherapy [34]. In that second study, the microdosing group was restricted to a “microdosing only” subsample (i.e., excluding participants combining microdosing with ADHD medication), yielding analyzed sample sizes of 180 at baseline, 50 at two weeks, and 38 at four weeks. In contrast, the TAU group comprised 37 at baseline, 27 at two weeks, and 28 at four weeks [34]. Demographically, both groups were similar in age (mid-to-late 30 s) and were predominantly based in Europe. Still, the TAU group was substantially more female-dominated than the microdosing cohort, and lifetime psychedelic experience was common in the microdosing group but uncommon in TAU [34]. TAU participants were required to be taking standard ADHD medication at baseline and to continue it during follow-up, and they were excluded if they used psychedelics during the study period; stimulant medications (methylphenidate- and amphetamine-class drugs) predominated, with smaller numbers using non-stimulants such as atomoxetine or guanfacine [34].

In contrast to these self-selected, remote microdosing cohorts, the phase 2A randomized clinical trial enrolled a clinically diagnosed adult ADHD sample under rigorous screening and safety procedures at two European outpatient sites (primarily University Hospital Basel, with a minor contribution from Maastricht) between December 2021 and December 2023 [36]. Participants were aged 18–65 years, had an established DSM-IV/DSM-5 ADHD diagnosis, and were required to have clinically meaningful current symptom severity at baseline (AISRS ≥ 26 and CGI-S ≥ 4), producing a sample characterized by substantial active impairment (mean baseline AISRS ~36; CGI-S ~4.8) [36]. Of 53 randomized participants (27 assigned to low-dose LSD and 26 to placebo), mean age was 37 years (SD 12), and 42% were female; nearly half were taking ADHD medication at screening before protocol-required washout, and recreational substance exposure histories were relatively common (e.g., lifetime cannabis and hallucinogen use reported by majorities). However, the current substance use disorder was excluded [36]. Retention was high relative to the naturalistic studies, with 46 of 53 completing the study period [36].

Finally, the ayahuasca retreat pilot drew from a markedly different population: a mixed community sample of adults attending a traditional ayahuasca retreat in Iquitos, Peru (January–May 2019), most of whom were not clinically diagnosed with ADHD [37]. Forty-nine participants completed ADHD symptom ratings pre- and post-retreat, with demographic data available for 47; ages ranged from 21 to 62 years (mean ~40.9), sex distribution was approximately balanced (23 male, 24 female; two unreported), and the sample was predominantly White with smaller representation of Hispanic, Black, Asian, and other ethnic categories [37]. Psychiatric history was self-reported and varied (including depression and anxiety). Still, only four participants reported an ADHD diagnosis, emphasizing that observed symptom changes primarily reflect shifts in self-reported ADHD-like symptoms in a largely non-ADHD-diagnosed retreat cohort rather than outcomes in a clinically confirmed ADHD sample [37]. Notably, the retreat setting enforced abstinence from prescribed medications on arrival, including ADHD pharmacotherapy, meaning symptom observations occurred in the absence of concurrent standard medication during ceremonies [37].

### 3.2. Psychedelic Interventions

Across the included studies, “psychedelic intervention” referred to markedly different exposure models, ranging from fully self-directed community microdosing to a fixed-dose, supervised low-dose LSD regimen delivered under randomized, placebo-controlled conditions, and to participation in a traditional ayahuasca retreat where the psychoactive exposure was embedded within a ceremonial program rather than standardized pharmacological dosing. In the prospective naturalistic microdosing studies, the psychedelic exposure was not assigned, supplied, or standardized by investigators; instead, participants were explicitly recruited because they independently intended to initiate microdosing as a form of self-management for ADHD symptoms, and the studies prospectively documented what they chose to take in real-world conditions over approximately four weeks [33,34,35]. Microdosing initiation occurred shortly after baseline assessment (typically 1–3 days later), and exposure data were gathered via daily reporting links and/or diary entries during the follow-up window, creating a naturalistic record of participants’ self-selected substances and doses while leaving core parameters—such as product source, exact preparation, potency, route of administration, and adherence to any specific dosing schedule—uncontrolled by design [33,34,35]. This lack of standardization was a defining feature of the intervention construct in these studies. It was treated by the authors as both ecologically valuable (capturing typical community practice) and interpretively limiting (introducing substantial heterogeneity in “dose,” timing, and fidelity) [33,34,35]. Finally, the ayahuasca retreat pilot differed fundamentally from the low-dose and microdosing studies in both intervention model and likely mechanism. Rather than administering repeated low or sub-perceptual doses, this study evaluated participation in a traditional ceremonial ayahuasca retreat in Iquitos, Peru, where participants attended approximately 3 to 11 ceremonies across retreats lasting from 8 days to 4 weeks. The ayahuasca brew was not chemically standardized, and the study did not report quantified dose levels of ayahuasca, DMT, or related alkaloids per session; therefore, a precise dose classification cannot be assigned. However, the intervention was clearly not a microdosing protocol and is most appropriately interpreted as repeated ceremonial, likely macrodose-style ayahuasca exposure embedded within a broader retreat context. This distinction is important because any observed effects may reflect a different pharmacological and contextual process than the low-dose LSD and self-directed microdosing studies, including acute psychoactive effects, ceremony-related factors, and repeated high-intensity experiential exposure rather than sub-perceptual repeated dosing [37].

Within these naturalistic microdosing cohorts, psilocybin-containing mushrooms or truffles were the predominant substances used, with smaller subsets reporting lysergamide microdosing (LSD and “novel lysergamides” such as 1P-LSD/ALD-52) and rare reports of other preparations [33,34,35]. Among participants who provided diary data (approximately half of the baseline cohort), roughly four out of five reported microdosing psilocybin/psilocin-containing material during the observation period, while fewer reported LSD or novel lysergamides, and only a single individual reported ayahuasca microdosing [33,34,35]. Reported mean quantities—available only for the diary-reporting subset—fell in the expected “microdose” range as practiced in the community, with psilocybin-containing material typically reported in the hundreds of milligrams (e.g., a mean in the ~700 mg range with wide variability), and lysergamide preparations reported in low micrograms (e.g., teens of micrograms for novel lysergamides and around a dozen micrograms for LSD in the subset providing data), again with substantial dispersion that is consistent with uncontrolled, participant-led dosing [33,35]. Some individuals switched substances during the follow-up period, further underscoring that the intervention was best conceptualized as “self-directed microdosing practice” rather than a single, uniform pharmacological exposure [33,35]. Because reporting completeness was limited and selective (only about half submitted daily dosing details), the available dosing descriptions likely represent an incomplete and potentially biased snapshot of exposure, and they cannot fully characterize the microdosing regimens of the full enrolled sample [33,34,35]. Importantly, these studies also tracked whether microdosing occurred alone or alongside ongoing first-line ADHD pharmacotherapy, operationalizing a “medication use alongside microdosing” characteristic to distinguish microdosing-only practice from combined use; however, the psychedelic exposure itself remained participant-driven in both subgroups [33,34,35].

A more structured—but still naturalistic—comparison framework was introduced in the second microdosing study package by contrasting a microdosing-only cohort with a “treatment as usual” group maintained on conventional ADHD medication without psychedelic use [34]. Here, the “psychedelic intervention” in the microdosing arm remained the same self-initiated practice documented via repeated assessments and daily reporting. Still, the design explicitly excluded participants who combined microdosing with ADHD medication from the microdosing arm used for the group comparison, thereby attempting to isolate a microdosing-only exposure against ongoing pharmacotherapy as the comparator condition [34]. The TAU group did not receive any psychedelic intervention, and participants were excluded if they reported psychedelic use during the follow-up interval; thus, the psychedelic exposure in this comparative study was defined primarily by participation in self-directed microdosing versus confirmed non-use in the medicated comparison group [34].

In contrast, the phase 2A randomized clinical trial evaluated a tightly specified low-dose LSD regimen intended to approximate a “microdosing-like” schedule while retaining clinical control, blinding, and standardized dosing [36]. Participants were randomized to receive either LSD or a placebo in a double-blind, parallel-group design, with a fixed dose of 20 µg LSD (as LSD base) administered twice weekly for six weeks, totaling 12 supervised dosing sessions [36]. LSD was delivered as a 1 mL oral drinking solution containing 29 µg LSD tartrate (equivalent to 20 µg LSD base) dissolved in 20% alcohol. In comparison, the placebo consisted of an identical 1 mL 20% alcohol solution without an active compound, supporting sensory matching and procedural equivalence across arms [36]. Dosing took place on-site under supervision throughout the six-week dosing phase; the first dosing day included enhanced monitoring and characterization, with participants remaining at the study center for approximately 6 h for clinical observation and acute effects/pharmacokinetic assessments.

In contrast, later dosing visits were designed to be less burdensome (participants could leave immediately after administration), balancing outpatient feasibility with safety oversight [36]. The trial also incorporated post-treatment follow-up assessments several weeks after the dosing period (around week 10), enabling evaluation of persistence of any symptom changes beyond the active dosing window [36]. Unlike the naturalistic microdosing studies, this regimen imposed uniformity of compound identity, dose, frequency, and administration context, while also requiring medication washout for drugs that could interact with LSD, thereby reducing—but not eliminating—sources of heterogeneity relevant to interpreting symptom change [36]. Notably, even under this “low-dose” protocol, acute subjective effects were measurable and, for some participants, sufficiently strong to contribute to discontinuation, highlighting that the tested dose sat toward the upper end of what many community users label “microdosing” and that the intervention may not have been fully subperceptual in this clinical sample [36].

Finally, the ayahuasca retreat pilot operationalized the psychedelic intervention as participation in a traditional ceremonial retreat program rather than a standardized pharmacological dosing regimen [37]. Participants attended retreats at a center in Iquitos, Peru, where ayahuasca was administered within a Shipibo ceremonial framework adapted for visiting participants; the intervention exposure therefore encompassed both ayahuasca ingestion and the broader retreat context, including preparatory restrictions and repeated ceremonies [37]. Retreat formats varied in duration (including approximately 8-day, 2-week, 3-week, and 4-week options), and the number of ceremonies available—and attended—varied accordingly, producing a range from roughly three to eleven ceremonies across participants [37]. The brew was not chemically standardized, and the authors emphasized that the psychoactive content likely varied across ceremonies due to natural variability in preparation, making dose consistency unassumed. The intervention could not be reduced to a fixed quantity of active constituents [37]. The retreat setting also implemented medical and psychiatric screening and enforced abstinence from medications and substances, including exclusion of attendees found to be taking prescribed medicines on arrival; participants were instructed to avoid illicit substances and follow dietary restrictions in the weeks before arrival, aligning with standard retreat safety practices and potentially shaping both the intervention experience and symptom reporting [37]. Thus, in this study, the psychedelic intervention was best conceptualized as a bundled exposure—multiple ayahuasca ceremonies within a structured retreat environment with preparatory and contextual constraints—rather than a controlled pharmacologic dose administered under clinical trial conditions [37].

### 3.3. ADHD Symptom Severity

ADHD symptom severity was the most consistently assessed clinical outcome across this body of work, although it was measured with different instruments and under markedly different exposure conditions. In the prospective naturalistic microdosing cohort, symptom change was tracked with the CAARS-S:SV (T-scores) at baseline, two weeks, and four weeks after participants began self-initiated microdosing, and the primary pattern was a pronounced, progressive reduction in self-reported symptoms over the first month [33]. The DSM-IV total symptom T-score (inattention plus hyperactivity/impulsivity) showed a significant time effect in mixed-model analyses, with symptom severity significantly lower at two weeks than baseline (mean reduction on the order of ten T-score points) and lower still at four weeks (mean reduction on the order of fifteen T-score points), with an additional significant decline from week two to week four, indicating an accumulating improvement rather than a rapid early plateau [33]. Parallel decreases were observed across CAARS-S:SV indices, including the ADHD index and the inattention and hyperactivity/impulsivity subscales; the inattention domain in particular started from very elevated baseline levels and moved downward over time, and by week four mean scores were below the “clinically elevated” threshold (T ≥ 65) on most reported indices, suggesting that, on average, participants shifted from clearly elevated symptom ranges toward more typical ranges on this screening measure during the observation window [33]. The study also explored whether conventional ADHD medication use alongside microdosing altered symptom trajectories and found a small but statistically detectable time-by-medication interaction concentrated at the two-week assessment: participants combining microdosing with prescribed medication tended to show a more minor early symptom reduction (and thus higher week-two symptom scores) than those microdosing without prescription, but this difference was not evident by week four, pointing to a potential delay or different early-course pattern rather than a stable attenuation of improvement [33]. Comorbidity alongside ADHD, while common, did not meaningfully moderate the symptom change trajectory; instead, comorbidity was more apparent as a marker of worse baseline status on some measures without clear evidence that it changed the direction of symptom improvement over four weeks [33]. Descriptively, a minority of participants met a non-response definition (no improvement or worsening), highlighting heterogeneity in individual trajectories even within the overall group-level improvement pattern [23].

The second study package reinforced the presence of symptom improvement in microdosing participants and, crucially, placed it in direct contrast to a “treatment as usual” group who continued conventional ADHD medication without psychedelics [34]. In that comparison, ADHD symptoms were again measured with CAARS-S:SV indices across the same baseline–two-week–four-week schedule. The defining result was a consistent time-by-group interaction across symptom domains: the microdosing-only group showed steeper symptom reductions over the month than the TAU group, and although microdosing participants began with higher inattention at baseline (consistent with TAU participants already being medicated at entry), by four weeks the microdosing group reported lower symptom T-scores than TAU across every CAARS-S:SV index (inattention, hyperactivity/impulsivity, DSM-IV total symptoms, and the ADHD index) [34]. In this naturalistic comparison, the microdosing-only cohort showed larger short-term reductions in self-reported symptom ratings than the TAU cohort [34], but because the groups were non-randomized, unblinded, and nonequivalent at baseline, this pattern should be interpreted as exploratory rather than as evidence that microdosing outperformed standard pharmacotherapy.

By contrast, the phase 2A randomized, double-blind, placebo-controlled trial—methodologically the strongest test in this set—did not demonstrate incremental symptom benefit of repeated low-dose LSD over placebo despite meaningful improvement in both arms [36]. Clinician-rated ADHD symptoms (AISRS) decreased from baseline to week six in both the LSD and placebo groups, but the between-group difference was not statistically significant and did not favor LSD on the primary endpoint; secondary symptom measures (including self-report scales such as CAARS domains and ASRS) followed the same overall pattern of within-group improvement without clear separation between LSD and placebo in the magnitude of change [36]. This divergence between naturalistic microdosing signals and the randomized trial result is especially salient because it suggests that short-term symptom improvement can occur in a structured treatment context even without pharmacologic advantage of LSD at the tested dose and schedule, underscoring the potential role of expectancy, repeated contact, and nonspecific trial participation effects in driving symptom change as measured by both clinician ratings and self-report [36].

Finally, the ayahuasca retreat pilot assessed ADHD-like symptoms using the ASRS before the first ceremony and the morning after the final ceremony, reporting significant pre–post reductions in inattention, hyperactivity/impulsivity, and total ASRS scores in a mostly non-diagnosed community retreat sample [37]. Although only a small minority self-reported an ADHD diagnosis, the overall retreat cohort showed substantial decreases across symptom domains, and these reductions were reported as not clearly dependent on retreat length or the number of ceremonies attended within the observed ranges [37]. Taken together, the symptom literature is internally inconsistent once study design is weighted. Uncontrolled naturalistic studies repeatedly report short-term reductions in self-rated ADHD symptoms after microdosing initiation or retreat exposure [33,34,35,37], but these signals arise under maximal risk of self-selection, expectancy, non-standardized exposure, regression to the mean, and selective retention. By contrast, the only randomized placebo-controlled ADHD trial found no statistically significant advantage of low-dose LSD over placebo on clinician-rated or self-reported ADHD outcomes [36]. Accordingly, the current evidence supports, at most, low-certainty associations between psychedelic use and perceived symptom improvement; it does not demonstrate drug-specific efficacy for core ADHD symptoms.

### 3.4. Emotional and Broader Psychosocial Functioning

Secondary outcomes were more consistently positive than core ADHD outcomes, but they should be interpreted as transdiagnostic correlates rather than evidence of ADHD-specific benefit. Across naturalistic microdosing cohorts, participants reported improved well-being, reduced expressive suppression, increased cognitive reappraisal, higher mindfulness scores, and lower neuroticism over approximately 2–4 weeks [33,34,35]. However, these studies were uncontrolled, self-selected, vulnerable to expectancy and attrition, and relied largely on self-report measures; accordingly, they provide low-certainty evidence of perceived change in affective/self-regulatory functioning rather than of change in core ADHD pathology.

The most reproducible secondary signals clustered in emotional well-being and self-regulatory style, not in objective cognition. Empathy findings were mixed, and the available cognitive signal was limited by the absence of consistent improvement in time-perception performance [33,34]. This pattern is important because improvements in emotion regulation, mindfulness, and negative emotionality may reflect changes in affective burden, stress reactivity, self-appraisal, or comorbid anxiety/depressive dimensions, any of which could secondarily influence ADHD symptom ratings without demonstrating a direct effect on attentional control, inhibitory dysfunction, or executive control.

Therefore, the secondary-outcome literature should not be read as confirming ADHD-specific efficacy. At most, it suggests that naturalistic psychedelic use may coincide with short-term improvements in adjacent transdiagnostic domains that are clinically relevant in many adults with ADHD, but that remain insufficiently separable from expectancy, context, and comorbidity in the current evidence base.

### 3.5. Cognitive Performance and Time Perception

Only one included study directly assessed a cognitive performance outcome alongside clinical symptom ratings, using an online auditory time reproduction paradigm intended to probe a cognitive domain that is often reported as atypical in ADHD—time perception and timing accuracy [33]. In this task, participants were presented with tones of fixed durations (1000, 1500, 3200, 3700, 5500, and 6000 ms) and were asked to reproduce each interval, producing a relative difference score in which values near zero indicate accurate reproduction, negative values reflect under-reproduction (underestimation), and positive values reflect over-reproduction (overestimation) [33]. The central finding was that initiating self-directed microdosing was not associated with a systematic improvement in timing performance over the 4-week observation window. Mixed-model analyses showed no main effect of time on the relative difference scores, indicating that, at the group level, performance did not reliably shift from baseline to 2 weeks or to 4 weeks after participants began microdosing [33]. This null effect stands in contrast to the contemporaneous reductions in self-reported ADHD symptoms and increases in well-being observed in the same cohort, but it should be interpreted cautiously. More specifically, the study did not detect improvement on this particular online auditory timing task over the four-week observation window; this should not be taken as evidence that cognitive effects were absent more broadly, because only one cognitive domain was assessed, and it was measured using a single remote paradigm [33].

Although there was no overall time-related improvement, the task demonstrated expected psychophysical structure across the sample: a strong main effect of interval length indicated that longer intervals tended to be reproduced less accurately than shorter ones, with a tendency toward greater underestimation as durations increased—an interval-dependent pattern commonly observed in timing research [33]. Notably, the absence of a time-by-interval interaction at the whole-sample level indicated that microdosing initiation did not meaningfully reshape this duration-dependent performance profile; participants’ relative accuracy across short and long intervals retained a similar pattern across baseline and follow-up assessments [33]. Thus, the data did not provide evidence of a change in this specific timing paradigm. Still, they are insufficient to exclude the possibility of cognitive effects in other domains or under more sensitive assessment conditions.

At the same time, this finding should not be overinterpreted as evidence against cognitive effects per se. The current evidence base has not yet made a robust attempt to characterize cognition in ADHD in relation to psychedelic use, because only one included study assessed only one cognitive domain using a single online auditory time-reproduction task over a relatively short follow-up period [33]. Time perception is relevant to ADHD, but it represents only one part of the broader cognitive phenotype, which also includes attentional control, inhibitory control, working memory, sustained performance, reward-related decision making, and executive regulation. Moreover, task sensitivity may have been limited by the naturalistic design, remote administration, psychophysical noise inherent to interval-reproduction paradigms, and the possibility that any pharmacological effects were subtle, transient, or expressed in domains not captured by this measure. Accordingly, the most balanced interpretation is not that cognitive effects were disproven, but that they have not yet been adequately tested in this literature and should be examined in future studies using broader, better-powered, and repeated neurocognitive assessments.

The only signal suggesting a subgroup-specific change emerged in an exploratory three-way interaction involving time, interval length, and concurrent conventional ADHD medication use during microdosing [33]. Specifically, among the subgroup reporting use of prescribed ADHD medication alongside microdosing, relative difference scores were higher at follow-up for the shortest (1000 ms) interval than for participants microdosing without medication, reflecting greater over-reproduction/overestimation of that short interval at both 2 and 4 weeks [33]. However, this interaction was interpreted cautiously for several reasons intrinsic to the dataset: the medicated subgroup at follow-up was small (on the order of the mid-teens at two weeks and single digits by four weeks), reducing stability of estimates, and the task itself used only two trials per interval, which can amplify measurement noise and make apparent differences more sensitive to outliers or transient fluctuations in attention during an online assessment [33]. Consequently, while the interaction suggests that medication status may have been associated with subtle, interval-specific shifts in timing behavior under naturalistic microdosing conditions, it does not provide strong evidence for a reliable cognitive enhancement effect and, if anything, points to a complex pattern that does not align straightforwardly with “improved” timing accuracy [33].

Overall, the cognitive-performance evidence in this review is limited to a single task and a single cohort. Still, the available data indicate that, over 4 weeks, self-directed microdosing was not associated with broad improvements in online time reproduction performance, despite concurrent self-reported improvements in ADHD symptoms and well-being [33].

### 3.6. Safety, Tolerability, and Intervention Acceptability

Safety and tolerability signals differed sharply by study type, with the most unmistakable and most systematically collected evidence coming from the phase 2A randomized clinical trial of repeated low-dose LSD. In contrast, the naturalistic microdosing and retreat studies provided only limited, indirect safety information because their designs prioritized symptom and psychosocial outcomes rather than structured adverse-event surveillance. In the randomized trial, participants received 20 µg LSD (base) or placebo twice weekly for six weeks under supervised outpatient administration, with medical and psychiatric screening, medication washout for potential interactions, and repeated safety monitoring (including vital signs, ECG, laboratory testing, urine drug screening, and suicidality assessment) [36]. Within this controlled context, no serious adverse events and no deaths were reported, and physiologic monitoring did not identify clinically meaningful between-group differences over time in electrocardiographic parameters; suicidality monitoring did not show newly occurring suicidal ideation during dosing [36]. At the same time, tolerability burdens were clearly higher with LSD than placebo: adverse events were more frequent in the LSD arm (124 total events) than in the placebo arm (64 total events), with treatment-related events more common under LSD and typical complaints including headache, nausea, fatigue, insomnia, and visual alterations [36]. Notably, the trial documented discontinuations during the dosing phase in both groups. Still, two discontinuations in the LSD arm were directly attributed to acute drug experiences described as strong or functionally impairing, illustrating that the selected “microdose-like” regimen was not uniformly subperceptual and could produce acute effects substantial enough to disrupt daily functioning for some participants [36]. This tolerability profile aligned with the study’s acute-effect characterization on dosing day 1, where subjective drug effects were detectably stronger under LSD than placebo, reinforcing the interpretation that 20 µg sits at the upper end of what many community users label microdosing and may be experienced as a mild but tangible altered state in a clinical ADHD population [36].

By contrast, the naturalistic online microdosing studies were not designed to assess safety as a primary or structured outcome. These studies followed self-initiated psychedelic use over approximately four weeks and focused mainly on symptom change, well-being, and psychosocial outcomes rather than on systematic adverse-event capture [33,34,35]. Although some dosing information was recorded in participant diaries, the reports do not describe a clinical-trial style safety framework such as predefined adverse-event collection, clinician-rated tolerability assessment, protocolized physiological monitoring, or formal adjudication of harms [33,34,35]. Accordingly, these studies should not be interpreted as providing safety data in the usual clinical sense; rather, they offer only limited indirect information from observational self-report designs.

The ayahuasca retreat pilot likewise did not provide a structured safety assessment. Although the retreat employed several screening and risk-management procedures—including psychiatric and medical screening, exclusion of minors and individuals with psychotic conditions and/or personality disorders, medication-related exclusions, and pre-retreat abstinence/dietary rules—the study’s outcomes focused on pre–post symptom change rather than systematic documentation of adverse physical or psychological events [37]. Therefore, the retreat study may be informative regarding the context in which exposure occurred, but it cannot be used to estimate adverse-event frequency, tolerability, or comparative safety.

Taken together, the only included study that systematically assessed safety and tolerability was the randomized clinical trial of repeated low-dose LSD delivered under clinical supervision [36]. In that setting, no serious adverse events were reported, but treatment-related adverse events were more frequent under LSD than placebo, and some participants discontinued because of acute drug effects [36]. In contrast, the naturalistic microdosing studies and the retreat-based ayahuasca study were not designed to assess safety systematically. They therefore cannot support meaningful conclusions about adverse-event incidence or comparative tolerability in real-world practice [33,34,35,37].

### 3.7. Risk of Bias Assessment

The bias risk assessment is presented in Table 3 (ROBINS-I) and Table 4 (RoB-2).

## 4. Discussion

The area of research on the use of psychedelics in treating ADHD is still developing. Only five experimental studies were included in this review, and their quality is, at best, average. However, this area is worth further exploration, as psychedelics are being intensively studied in many other neuropsychiatric disorders. It can be expected that, over time, more studies will be conducted. Below, the findings from the included studies are discussed and placed in the broader context of psychedelic research.

### 4.1. Summary of the Main Findings (From the Present ADHD-Focused Evidence Base)

Across the five included studies, the highest-informational-value evidence is the randomized, double-blind, placebo-controlled trial of repeated low-dose LSD, which did not demonstrate superiority over placebo on clinician-rated or self-reported core ADHD outcomes. By contrast, the positive signals in the remaining studies derive predominantly from naturalistic, self-selected, non-standardized, and largely self-report designs, three of which examined closely related microdosing cohorts. Accordingly, the evidentiary center of gravity of the present review should not be the consistency of uncontrolled symptom improvement, but the contrast between repeated naturalistic improvement and the absence of clear drug–placebo separation under controlled conditions. In a large meta-analysis examining placebo response and its predictors in ADHD, placebo-associated symptom improvement was robust. It appeared to increase markedly over the last two decades, complicating the interpretation of short-term symptom changes when expectations are high [38].

This hierarchy is critical for interpretation. The naturalistic studies are useful for hypothesis generation and for identifying domains in which participants report change, but they do not provide robust evidence of drug-specific efficacy for adult ADHD. Their results are highly vulnerable to self-selection, expectancy, reporting bias, heterogeneous exposure conditions, attrition, and non-equivalent comparison structures. Therefore, repeated short-term improvement in uncontrolled settings should be interpreted as a signal of perceived benefit under real-world use conditions, not as confirmatory evidence that psychedelics improve core ADHD pathology.

An additional interpretive possibility that should be acknowledged is that any pharmacological benefit of psychedelics in ADHD may be predominantly acute rather than cumulative. In the controlled low-dose LSD trial included in this review, the main efficacy analyses focused on change across repeated dosing over several weeks, and therefore primarily tested whether LSD produced a superior cumulative trajectory relative to placebo. However, such a design may miss transient drug-related effects occurring on or shortly after dosing days. In other words, it is possible that both study arms improved over time due to non-pharmacological influences such as expectancy, repeated clinical contact, symptom monitoring, or regression to the mean, while the assessment schedule did not adequately capture a short-lived pharmacological effect of LSD, or endpoints that emphasized cumulative change. This issue may be especially relevant in ADHD, where clinically meaningful changes could occur in attention, arousal, task engagement, or emotional regulation during the acute post-dose window without necessarily accumulating into a large between-group difference over weeks. Future trials should therefore assess both acute and cumulative effects, using repeated measurements on dosing days and in the immediate post-dose period in addition to longer-horizon outcomes.

Within the naturalistic microdosing literature, self-reported ADHD symptom scores, well-being, and affect-related measures often improve together over short follow-up periods. However, this pattern should not be taken to indicate selective improvement in core ADHD mechanisms. A more conservative interpretation is that these studies capture a broad cluster of perceived changes spanning mood, self-appraisal, coping style, and everyday functioning, any of which may secondarily influence how participants rate their ADHD symptoms.

This distinction is particularly important for the secondary outcomes reported in the present review. Improvements in emotion regulation, mindfulness-related capacities, and neuroticism-like traits should not be interpreted as equivalent to improvement in core ADHD pathology. These domains are transdiagnostic, are strongly shaped by comorbid anxiety/depression and stress burden, and may reflect change in affective dysregulation, self-reflective style, or contextual adaptation rather than direct change in attentional control, inhibitory control, or executive dysfunction. Accordingly, the current evidence is more compatible with possible effects on adjacent affective/self-regulatory dimensions than with demonstrated efficacy for core ADHD symptoms. Trait mindfulness increases have been documented after psilocybin, with evidence linking post-acute increases in mindfulness to the intensity/quality of the acute experience and, in some work, to serotonergic receptor-level changes [39,40,41]. A key point for interpretation is that such changes are not unique to ADHD samples; they appear in multiple populations and designs, which makes them plausible “bridge mechanisms” for why participants might endorse fewer ADHD symptoms over time (improved emotional labeling, reduced self-judgment, less avoidant coping), even if objective attention metrics remain relatively stable.

Relatedly, the ADHD-focused naturalistic microdosing studies in this review reported decreases in neuroticism over the observation window, while other personality domains appeared comparatively stable. This direction of change aligns with a broader multi-year literature suggesting that psychedelic experiences can be associated with reduced neuroticism and sometimes increased openness. However, durability and boundary conditions remain debated [42,43]. Importantly, controlled, mechanistic work indicates that these longer-lasting trait shifts may depend on the subjective intensity/meaningfulness of the acute experience. This observation matters for microdosing because low doses can sometimes produce noticeable acute effects (and therefore expectancy cues) without necessarily making the same depth of “peak” experience as higher-dose, supported therapy models [32,43].

At the same time, the ADHD-focused evidence base in this review also contains a notable negative/limited cognitive signal, exemplified by the absence of consistent improvement in an online time-perception task. This is broadly compatible with the wider microdosing literature: controlled studies and meta-analytic syntheses generally find weak or inconsistent objective cognitive benefits of microdosing and emphasize that some effects (when present) are minor, domain-specific, or even suggest slight impairments in certain control-related functions [32,44]. This broader pattern matters because it helps contextualize the ADHD findings: the most reliable “movement” across outcomes tends to occur in subjective well-being, affective appraisal, and self-regulatory style, rather than in robust improvements on objective cognition.

Finally, when the ADHD-focused evidence is placed into the larger microdosing and psychedelic-trials context, the central interpretive constraint is that expectancy and masking are persistent methodological challenges. The best-known microdosing placebo-control demonstration—the self-blinding citizen-science study—showed that many reported microdosing benefits could be accounted for by placebo-related processes and breaking the blind [30]. A recent review specifically assessing microdosing concludes that the current evidence base remains insufficient to determine whether microdosing benefits are pharmacologically specific or largely placebo- or context-driven [29]. And systematic reviews focusing on psychedelic trial design highlight that masking and placebo control are recurrent weaknesses across decades of psychedelic RCTs, reinforcing that symptom-scale improvements—especially over short time frames—must be interpreted cautiously when blinding is fragile [45,46,47].

In summary, the present ADHD-focused evidence base supports a restrained interpretation. First, uncontrolled naturalistic psychedelic use is repeatedly associated with short-term improvements in self-reported ADHD symptoms and broader psychosocial well-being. Second, the most consistent accompanying changes occur in transdiagnostic affective and self-regulatory domains rather than in objective cognition or clearly demonstrated core ADHD mechanisms. Third, when methodological rigor is increased through placebo control, standardized dosing, and blinded assessment, the apparent drug-specific signal becomes weak or undetectable. The current evidence therefore supports, at most, low-certainty associations with perceived improvement, not established ADHD-specific efficacy.

### 4.2. Interpreting the Discrepancy: Why Naturalistic Microdosing Looks Strong While the RCT Does Not

The simplest way to reconcile the “large” naturalistic microdosing signal with the RCT’s lack of drug–placebo separation is to treat them as measuring two different things: naturalistic studies are exquisitely sensitive to contextual therapeutic ingredients (expectancy, motivation, lifestyle change, community narratives, regression to the mean, and selective retention), whereas placebo-controlled trials are designed—imperfectly, but deliberately—to subtract those nonspecific influences and isolate incremental pharmacologic benefit. In ADHD specifically, the background placebo response is already substantial. It has increased over time in medication trials, making it easier for any highly anticipated new approach to look dramatic in uncontrolled settings. A large meta-analysis of ADHD placebo arms (94 trials; >6000 participants randomized to placebo) reported substantial placebo improvement and concluded that placebo response in ADHD trials increased markedly from 2001 to 2020 (with regional differences such as higher placebo response in the US) [38]. If symptom ratings are the primary endpoint—as they typically are in short studies—then any intervention wrapped in high expectation, frequent self-monitoring, and a compelling cultural narrative can yield sizable pre–post change even when the drug-specific effect is small or absent. At present, the ADHD literature does not permit a confident decomposition of observed benefit into drug-specific effects, expectancy effects, self-monitoring effects, or broader setting- and ritual-related influences.

One plausible interpretation of this discrepancy is that the improvements observed in naturalistic studies may be driven in part by non-pharmacological influences, including expectancy, repeated self-monitoring, and other contextual factors. In contrast, the placebo-controlled trial was better positioned to isolate incremental drug-specific effects. This does not mean that the naturalistic improvements were unreal, but rather that they cannot be assumed to reflect pharmacological efficacy alone. More fundamentally, the current evidence does not allow the active components of apparent benefit to be disentangled with confidence. Any observed improvement could reflect direct pharmacological action, the psychological and behavioral consequences of entering an altered state of consciousness, contextual and psychotherapeutic ingredients such as preparation, ritual, support, and integration, or more nonspecific forms of psychological activation, including hope, salience, self-monitoring, and renewed goal engagement. These pathways are not mutually exclusive, and in real-world psychedelic use, they are typically tightly interwoven. However, the existing ADHD-focused literature is not designed to permit a clear decomposition of these components. This epistemic limitation should temper causal interpretation: even when participants report meaningful improvement, the present evidence cannot establish which part of that change is drug-specific, which is state-dependent, and which is context- or expectancy-mediated.

Stated plainly, the present ADHD literature does not allow one to separate pharmacological effects of the psychedelic compound from expectancy, self-monitoring, therapeutic framing, ritual/setting, and other contextual influences; therefore, claims of ADHD-specific drug efficacy are premature.

For the present review, this broader microdosing literature is informative mainly as a methodological comparator: it suggests that favorable naturalistic reports in ADHD should be interpreted cautiously, because in adjacent low-dose psychedelic research the apparent benefits commonly attenuate when placebo control, masking, and standardized dosing are introduced.

### 4.3. Why Self-Reported ADHD Improvement May Not Imply Direct Cognitive Benefit

A more proportionate interpretation of the included studies is not that psychedelics have been shown to improve core ADHD neurocognition directly, but that changes in psychological functioning may help explain why some participants reported lower ADHD symptom burden. In adults with ADHD, impairment is shaped not only by attentional and executive difficulties, but also by emotion dysregulation, stress reactivity, frustration tolerance, avoidance, and other self-regulatory processes that influence how symptoms are experienced and reported [48,49,50,51,52]. From that perspective, improvements in emotional regulation or self-related functioning could plausibly reduce day-to-day impairment and subjective symptom severity even without demonstrating a direct effect on underlying cognitive mechanisms.

A further interpretive complication is the heterogeneity of the populations included in this review. The evidence base combines a clinically diagnosed adult ADHD sample studied under randomized controlled conditions with self-selected community participants who either reported an ADHD diagnosis or met screening thresholds for elevated ADHD-like symptoms in online or retreat contexts. Across the naturalistic studies, psychiatric comorbidity was common and not consistently controlled in a way that would allow clear separation of core ADHD change from change in affective burden. This matters because improvements in well-being, neuroticism, emotional regulation, and related self-regulatory constructs may reflect modulation of depression-, anxiety-, or stress-related comorbidity rather than a direct effect on the core neurocognitive domains of ADHD. Accordingly, reductions in self-reported ADHD symptoms should not automatically be interpreted as evidence that psychedelic exposure targeted the disorder’s primary attentional or executive mechanisms.

This distinction is relevant because the studies in this review, particularly the naturalistic ones, relied heavily on self-report outcomes and, in some cases, observed changes in well-being, emotion-related processes, and related psychological domains alongside reduced ADHD symptom ratings. A similar logic is seen in the broader ADHD literature, where mindfulness-based interventions can improve symptoms and functioning. However, these benefits are often stronger relative to waitlist controls than to active comparators, suggesting effects on coping, awareness, and self-regulation rather than a direct normalization of core neurocognitive deficits [53,54,55]. Accordingly, psychological improvement may still be clinically meaningful even if it does not imply a stimulant-like or disorder-specific cognitive effect.

Related constructs such as self-compassion, reduced self-criticism, and lower perseverative negative thinking may also be relevant to how adults with ADHD evaluate their functioning. Adults with ADHD tend to report lower self-compassion and greater perceived criticism, and these factors are associated with poorer mental health [56,57]. In parallel, psychedelic studies outside ADHD have reported changes in self-compassion, affect regulation, rumination, and thought suppression [58,59]. However, in the context of the present review, such findings should be treated only as suggestive analogies rather than evidence of established mechanisms in ADHD. Overall, the available data support only a cautious conclusion: psychological or self-regulatory changes may help explain self-reported symptom improvement, but the current evidence is insufficient to determine whether these shifts are pharmacological, non-pharmacological, or the product of both.

### 4.4. The Cognitive Null Result: Time Perception and What It Implies

A notable feature of the ADHD-focused findings is that time perception did not improve alongside self-reported symptoms and well-being. This matters because timing difficulties, often described clinically as “time blindness,” have long been considered relevant to ADHD. Meta-analytic and experimental work suggests that people with ADHD often show deficits across timing tasks. Still, these effects are heterogeneous and depend on task structure, interval duration, modality, and the extent to which tasks recruit attention, working memory, and motivation [60,61,62,63,64,65]. Thus, a null effect on an online time reproduction task does not necessarily contradict ADHD timing theory; rather, it suggests that the reported benefits may not have been mediated by basic interval timing, or that the measure used was not sensitive to the most relevant timing-related differences.

More broadly, the null cognitive result suggests that improvements in ADHD symptoms may occur without measurable gains in timing performance. This is consistent with models proposing that functional improvement may be driven more by changes in mood, stress reactivity, and self-regulation than by changes in core neurocognitive processes. Timing deficits in ADHD are thought to reflect multiple interacting mechanisms, including executive dysfunction, delay aversion, reward sensitivity, and dopaminergic differences [66,67,68,69]. In this context, a short naturalistic intervention may plausibly improve perceived functioning while leaving timing performance unchanged.

Methodological factors may also have contributed. The sensitivity of time reproduction tasks depends strongly on design features such as trial count, interval sampling, modality, and testing context, and reliability is only fair to good in some paradigms [70,71]. More generally, cognitive measures with few trials have limited power to detect subtle change, especially in remote testing settings [72,73]. This is particularly relevant because interval reproduction is shaped by well-known psychophysical effects, including greater under-reproduction at longer intervals and central tendency biases, which can mask small intervention-related shifts [71,74,75].

Placed alongside the broader psychedelic literature, this null result is also informative. Low-dose LSD has been associated with temporal dilation in healthy samples, and full-dose psychedelic studies have reported altered timing and temporal control [74,76,77]. The absence of such effects here may reflect a mechanistic mismatch: any ADHD-related benefits may have been more affective or self-regulatory than cognitive, while timing performance may have been too dependent on attention, motivation, and remote-task variability for small pharmacological effects to emerge clearly [67,69,70,78].

More generally, the absence of consistent objective cognitive improvement across the included evidence weakens any strong claim that the observed changes preferentially target core ADHD domains. At present, the available studies more convincingly support the possibility of changes in subjective distress, emotional functioning, or perceived self-regulation than they do a demonstrable improvement in executive or attentional performance specific to ADHD. designs, potentially obscuring any minor pharmacologic effects that controlled lab paradigms can reveal. Taken together, the cognitive null result usefully narrows the interpretive space. It suggests that the symptomatic improvements observed in the present ADHD-focused evidence do not require (and did not clearly coincide with) a measurable correction of interval timing—supporting the view that short-term benefit may be driven more by affective and self-regulatory processes than by direct enhancement of ADHD neurocognition. At the same time, because timing measures are heterogeneous and design-sensitive, the null should not be treated as definitive evidence that timing is unaffected; instead, it highlights the need for future work to use more reliable, higher-trial cognitive timing batteries, ideally paired with motivational manipulations (e.g., reward) and comparator conditions, to determine whether any timing-related change is present but currently under-detected.

### 4.5. Ayahuasca Retreat Findings: Why “Set and Setting” May Be Doing Most of the Work

The ayahuasca retreat symptom reductions observed in the present ADHD-adjacent evidence are best interpreted through the lens of set and setting, because retreat participation is not a “drug-only” exposure; it is a bundled intervention in which pharmacology is embedded within a highly structured social, cultural, and behavioral ecology. Contemporary psychedelic theory has increasingly treated these extra-pharmacological factors as active ingredients rather than mere confounds. Hartogsohn’s framework explicitly links psychedelic effects to placebo science, arguing that expectations, meaning, ritual, and social cues can shape not only acute experience but also downstream clinical outcomes [79,80]. Methodological reviews of psychedelic trials similarly emphasize that context variables (preparation, therapeutic support, environment, music, rapport, integration) can substantially influence both subjective effects and measured symptom change—so much so that isolating pharmacology requires meticulous design [81,82]. In this context, it is unsurprising that a retreat setting—often characterized by high expectancy, strong ritual framing, intensive introspection, and social support—could yield extensive pre–post improvements on symptom scales even when the causal pathway is primarily contextual rather than pharmacologically specific.

Empirical work in ayahuasca populations supports the claim that ceremonial and support features predict mental health outcomes. An extensive international survey analyzing ayahuasca drinkers reported associations between mental health/wellbeing outcomes and contextual factors such as ceremonial practices, motivations, and additional supports, suggesting that “how” ayahuasca is taken (ritual structure, perceived safety, guidance, integration practices) is tightly linked to reported benefits [83]. Longitudinal observational studies of facilitated ayahuasca use likewise show improvements in mental health and wellbeing over time in retreat-like settings, reinforcing that real-world benefits are often observed—but also that these benefits occur in a highly confounded package of pharmacology plus context, selection, and behavior change [84,85]. Notably, at least one naturalistic study has explicitly attempted to disentangle ayahuasca’s pharmacologic contribution from retreat context using placebo control within retreat groups, directly acknowledging that expectation and setting can account for a meaningful portion of apparent “ayahuasca effects” [86,87]. This is a crucial point for interpreting retreat-based ADHD-like symptom improvements: if mental health indices can shift substantially under placebo within the same ceremonial container, then large pre–post deltas in uncontrolled retreat studies cannot be assumed to reflect drug-specific efficacy.

Several specific retreat components plausibly drive symptom improvement through well-established psychological and behavioral pathways that are relevant to ADHD-like complaints. Retreat participation often involves temporary removal from occupational overload, reduced digital distraction, consistent daily routines, and strong behavioral constraints (e.g., diet, abstinence from substances/medications). Even independent of psychedelics, such changes can improve sleep regularity, stress physiology, and perceived attentional control. In ayahuasca settings, these contextual drivers are not incidental; they are typically framed as part of the healing model and may amplify expectancy and adherence. The broader psychedelic literature shows experimentally that even seemingly “peripheral” setting elements—such as music—can shape acute experience and possibly outcomes; for example, work manipulating musical genre in psilocybin sessions highlights that setting characteristics can alter subjective trajectories in therapeutically consequential ways [82,86]. From this perspective, a retreat’s structured ritual (songs/icaros, ceremonial pacing, facilitator guidance) can be conceptualized as an engineered setting intervention that scaffolds emotion processing, catharsis, and meaning-making—processes that can reduce distress and improve self-regulation without necessarily changing core neurocognitive deficits.

The retreat literature also increasingly shows that relational and integrative supports—the human container around the drug—predict outcomes. Reviews and empirical analyses in psychedelic-assisted therapy indicate that therapeutic alliance, rapport, and pre-session relational variables influence the quality of acute experiences (including emotional breakthrough) and are associated with subsequent clinical improvement [88,89,90]. New measurement work aimed at quantifying “set” (mindset/intention) and “rapport” demonstrates that these predictors can explain substantial variance in mystical- and breakthrough-type experiences under controlled administration, underscoring that person-and-context factors are not noise; they are mechanistically upstream of the very experiences often linked to therapeutic change [91]. Retreats usually provide an intense version of these ingredients: group cohesion, trusted guides, shared narratives, and post-ceremony sharing circles can function as a high-dose psychosocial intervention. When symptom outcomes are self-reported shortly after such a concentrated psychosocial experience, it is plausible that a significant fraction of measured improvement reflects changes in appraisal, hope, self-efficacy, and emotion regulation—dimensions that ADHD symptom scales are sensitive to.

Finally, retreat effects may be amplified by how difficult experiences are handled. Challenging psychedelic experiences are everyday, and emerging work suggests that strategies for navigating difficulty—often taught implicitly through preparation and guidance—relate to emotional breakthrough and longer-term benefit [92]. Retreats typically include culturally embedded “scripts” for interpreting challenge (purging as cleansing; fear as something to surrender to), which can transform acute distress into a meaningful narrative of progress, thereby strengthening expectancy and perceived benefit. Prospective ecological and retreat-based studies also point to post-retreat changes in affect and mindfulness-like skills in daily life, consistent with the idea that the retreat container can catalyze improvements in emotional regulation and metacognitive awareness—again, pathways that can reduce ADHD-like impairment even without directly enhancing attention or timing mechanisms [87,93].

In sum, the most defensible interpretation of the ayahuasca retreat findings is that they demonstrate the potency of a multicomponent intervention—psychedelic pharmacology plus ritual, expectancy, behavioral restructuring, social support, and integration—rather than isolating ayahuasca as a targeted ADHD treatment. The broader literature repeatedly shows that contextual variables are predictive, experimentally influential, and methodologically central in psychedelic research, making it likely that “set and setting” account for a substantial share of retreat-associated symptom change.

### 4.6. ADHD-Specific Safety and Ethical Considerations

Safety remains an important concern in this literature, but the present review provides only limited ADHD-specific evidence on this question. Among the included studies, structured safety and tolerability data were available primarily from the supervised low-dose LSD trial. In contrast, the naturalistic microdosing studies and the retreat-based ayahuasca study did not systematically assess adverse events. Accordingly, conclusions about safety in ADHD cannot be drawn directly from the current ADHD-focused evidence base and must remain cautious.

What is specifically relevant to ADHD is that risk appraisal in this population is unlikely to mirror that of healthy-volunteer psychedelic studies. Adults with ADHD commonly present with psychiatric comorbidity, including anxiety, mood disturbance, sleep problems, and substance-use vulnerability, and many also use concurrent medications such as stimulants, atomoxetine, antidepressants, or anxiolytics. These factors may influence both acute tolerability and longer-term psychiatric risk, and they complicate the interpretation of uncontrolled or naturalistic exposure. In that respect, broader psychedelic safety reviews remain relevant, but mainly as indirect context rather than condition-specific evidence. Recent syntheses suggest that, under structured research conditions with screening and supervision, serious adverse events in classic psychedelic studies are uncommon. However, transient acute adverse effects and limitations in adverse-event reporting remain important concerns [94,95]. The key implication for ADHD is therefore not reassurance, but uncertainty: the field still lacks adequate data on how these risks may differ in a clinically complex, often medicated population.

One safety issue of particular relevance in ADHD-oriented settings is psychiatric vulnerability. A recent synthesis of psychedelic-induced psychosis found very low incidence estimates overall in population and controlled-study contexts, but substantially higher risk when individuals with psychotic disorders are included, supporting the routine exclusion of those with personal or family psychosis-spectrum risk in clinical trials [96]. In parallel, a systematic review of case reports concluded that psychedelic exposure can, in some cases, be linked to schizophrenia-spectrum psychosis and affective destabilization, including bipolar presentations [97]. This point is not unique to ADHD, but it has practical salience here because differential diagnosis, psychiatric comorbidity, and family- history assessment are central parts of adult ADHD evaluation. Any future ADHD-focused psychedelic trial should therefore apply careful screening for psychosis- and bipolar-spectrum vulnerability.

Medication interaction is another issue with particular relevance to ADHD populations. A review of serotonin toxicity risk concluded that classic serotonergic psychedelics alone are unlikely to produce severe serotonin syndrome,. butStill, the assessment becomes more complicated when psychedelics are combined with other serotonergic agents or with monoamine oxidase inhibition [98]. This is especially pertinent for ayahuasca, whose harmala alkaloids introduce MAOI-related interaction concerns, and for adults with ADHD who may be taking antidepressants or other psychotropic medications. Related reviews on concomitant antidepressant use likewise emphasize the need for medication reconciliation and conservative protocols, both because co-use may alter safety and because it may complicate interpretation of psychedelic effects [99]. Thus, medication status is not a peripheral issue in ADHD-focused research but a core design and safety variable.

Ethical considerations are also best understood here through an ADHD-specific lens. Psychedelic interventions involve altered perception, heightened emotional lability, and increased suggestibility during the acute state, which can complicate autonomous, ongoing consent and increase vulnerability within therapeutic or quasi-therapeutic settings. Ethics analyses and professional guidance in psychedelic treatment have therefore emphasized strengthened informed consent, clear boundaries, oversight, and robust response pathways for misconduct [100,101,102]. In the context of ADHD, these safeguards may be especially important because impulsivity, emotional dysregulation, novelty seeking, and difficulties with long-range risk appraisal could affect how individuals evaluate self-directed use, retreat participation, or informal sourcing. These concerns are amplified in retreat settings, where regulatory oversight may be limited, accountability structures may be ambiguous, and the possibility of exploitation or abuse has been sufficiently recognized to motivate dedicated guidance within ayahuasca communities [103,104].

The safety picture that emerges when psychedelic interventions are delivered under modern research conditions is broadly consistent across substances and indications: in screened participants, within structured protocols, serious adverse events are uncommon, while transient acute adverse effects are expected and require active management. An extensive recent systematic review and meta-analysis in JAMA Psychiatry (114 studies; 3504 participants) found that serious adverse events were rare in classic psychedelic research and that serious events were primarily observed in participants with preexisting neuropsychiatric conditions; the authors also highlighted widespread limitations in adverse-event detection and reporting practices across studies [94]. A separate 2024 meta-analysis focusing on safety/risk assessment similarly concluded that classic psychedelics are generally well-tolerated in controlled settings, but with predictable acute-phase effects such as elevated heart rate/blood pressure and nausea, alongside common complaints like headache and anxiety [95]. These findings support a nuanced clinical position: “physiologically low lethality” is not the same as “low-risk,” because tolerability depends heavily on screening, dose control, supervision, and the ability to intervene during acute distress. 

That distinction becomes more critical when translating findings to real-world use (including self-directed microdosing and retreat contexts), where dose uncertainty, polysubstance use, and variable supervision increase risk. Even in clinical research, classic psychedelics can acutely increase autonomic arousal (blood pressure and heart rate). They can precipitate panic, agitation, or dysphoric reactions that—while usually time-limited—can be clinically significant if not contained [94,95]. In ADHD populations, these considerations interact with common comorbidities (anxiety, mood disorders, substance-use history) and with concurrent medications (stimulants, antidepressants), making risk appraisal more complex than in healthy-volunteer studies. 

A central safety question in ADHD-relevant contexts is the risk of psychiatric destabilization, particularly psychosis-spectrum outcomes in vulnerable individuals. A large synthesis reconsidering psychedelic-induced psychosis across multiple study types reported very low incidence estimates in population and controlled-study contexts overall, but substantially higher risk when individuals with psychotic disorders are included—an essential justification for the widespread exclusion of people with personal or family psychosis risk in clinical trials [96]. Complementing incidence estimates, a systematic review of case reports in Psychological Medicine concluded that credible case evidence links psychedelic use to schizophrenia-spectrum psychosis and affective destabilization (including bipolar presentations), underscoring that rare but severe adverse psychiatric outcomes do occur and are clinically meaningful even if they are infrequent in screened trials [97]. The practical implication for ADHD is ethical as much as medical: any consideration of psychedelic exposure should explicitly address psychosis and bipolar vulnerability (personal history and first-degree family history) because the downside tail risk is high.

Another risk domain with outsized ethical relevance is Hallucinogen Persisting Perception Disorder (HPPD) and related persisting perceptual changes. A systematic review of published HPPD case descriptions cataloged a broad range of visual phenomena and emphasized heterogeneity in symptom profiles and severity [105]. A more recent systematic review of pharmacological treatment approaches concluded that no single clearly effective therapy can be identified from existing evidence, which remains dominated by case reports and small series—meaning that for a subset of affected individuals, the condition can be challenging to treat and prolonged [106]. This matters ethically because informed consent must cover not only likely short-term discomforts (nausea, headache, anxiety) but also low-probability, potentially persistent outcomes that can impair daily functioning and may not have a reliable remedy.

Drug–drug interactions add another layer that is especially salient for ADHD, where polypharmacy is common (stimulants, atomoxetine, antidepressants, anxiolytics). A widely cited review on serotonin toxicity risk with serotonergic psychedelics argues that classic psychedelics alone are unlikely to produce severe serotonin syndrome. Still, that risk evaluation becomes more consequential when combined with other serotonergic agents and especially with MAO inhibition (a key issue for ayahuasca, which contains MAO-inhibiting harmala alkaloids) [98]. Related clinical guidance documents and emerging reviews discuss interaction risks and the reality that antidepressant co-use may attenuate psychedelic effects or complicate safety assessment, reinforcing the need for medication reconciliation and conservative protocols rather than informal “stacking” [107]. For retreat-based ayahuasca in particular, the MAOI component makes interaction screening ethically non-negotiable, because combining MAO inhibition with certain antidepressants or other agents can plausibly elevate toxicity risk.

Ethically, psychedelic interventions pose distinctive challenges because they involve heightened suggestibility, emotional lability, and altered reality testing during the acute state—conditions that can compromise ordinary assumptions about autonomous, ongoing consent. A detailed ethics analysis of informed consent in psychedelic-assisted psychotherapy highlights that the unpredictability of the psychedelic experience can impair a patient’s ability to advocate for themselves in-session, and that this vulnerability increases the importance of advance discussion, boundary clarity, and oversight [100]. A 2025 scoping review of ethical issues in psychedelic-assisted treatments similarly identifies patient vulnerability as a core ethical concept. It links it to concrete risks: physical/psychological harm, setting-related hazards, and regulatory/oversight gaps [101]. Professional-practice guidelines published in 2023 emphasize strengthened informed consent procedures, explicit boundary and dual-relationship prohibitions, and robust reporting/response pathways for misconduct—reflecting a field-wide recognition that ethical safeguards must evolve alongside efficacy evidence [102].

These ethical concerns are not abstract. The psychedelic field has faced high-profile scrutiny over trial conduct and safeguards, especially in the MDMA-assisted therapy space, where regulators and independent commentators have raised concerns about bias, functional unblinding, and misconduct within trials, including documented cases of therapist abuse. Regardless of one’s interpretation of MDMA’s efficacy data in PTSD, the episode has sharpened the ethical baseline for psychedelic work: when participants are in an unusually vulnerable state, the duty to prevent exploitation and to ensure data integrity is higher, not lower.

Finally, retreat contexts (including ayahuasca tourism) intensify ethical complexity because they frequently operate outside medical regulatory frameworks while serving individuals who may be psychologically vulnerable. Scholarly work on globalized ayahuasca practices discusses recurring ethical tensions, including power differentials, ambiguous accountability structures, and the problem of sexual misconduct in ceremonial economies [103]. Related work reflecting on the development of community guidelines for preventing sexual abuse in ayahuasca settings illustrates that these harms have been salient enough to motivate formalized prevention guidance—an implicit acknowledgement that “spiritual” settings do not protect participants from exploitation [104]. In addition, ayahuasca tourism raises distributive and cultural ethics questions—about sustainability, commodification of Indigenous knowledge, and extractive dynamics—that do not typically arise in conventional pharmacotherapy discussions but are directly relevant when retreat participation becomes a quasi-clinical pathway for people seeking help.

In sum, the responsible interpretation of the ADHD-adjacent psychedelic evidence requires holding two truths at once: (1) under rigorous screening, dosing control, and supervision, severe adverse outcomes in trials appear relatively uncommon, while acute adverse effects are common but usually manageable; and (2) outside those conditions, risks scale nonlinearly due to interaction hazards, dose uncertainty, psychiatric vulnerability, and ethical exposure to coercion or abuse, with particular relevance for populations likely to have comorbidity and polypharmacy such as adults seeking ADHD symptom relief.

### 4.7. Implications for Future Research (Design Targets)

ADHD-focused psychedelic research should move beyond diagnostically broad samples and adopt endophenotypic or mechanistically anchored stratification. This is important because ADHD is phenotypically heterogeneous, and apparent treatment effects may reflect changes in partially separable domains such as cognitive control, response-time variability, reward sensitivity, emotional dysregulation, or sleep/circadian instability rather than a unitary change in “ADHD” as a whole.

Future ADHD-focused psychedelic research needs to be designed as if the main scientific problem is not “does it work?” but “can we separate pharmacology from expectancy, context, and measurement artifacts in a condition with strong placebo responsiveness?” Placebo response in ADHD trials is substantial and has been shown to increase over time, which means short-horizon symptom-scale improvements are intrinsically ambiguous unless expectancy is measured and controlled [38]. The microdosing field already provides a concrete warning: the largest placebo-controlled microdosing experiment to date found broad improvements over time, but little evidence of robust between-group differences, with belief about having taken a microdose tracking outcomes [30]. Reviews of controlled microdosing studies similarly conclude that repeated low-dose LSD shows acute effects but limited evidence for sustained mood/cognition benefits and emphasize that perceptibility and expectancy are central threats to inference [31]. Taken together with critiques of blinding in psychedelic RCTs—which argue that effect sizes are likely inflated by unblinding and high response expectancy—ADHD trials in this space must treat masking and expectancy quantification as primary endpoints rather than optional add-ons.

A first design target is therefore better masking. Methodological guidance for psychedelic trials repeatedly recommends active placebos, dose–response designs, and improved reporting of blinding success. Recent work specifically reviewing active placebo options proposes criteria for an “ideal” psychedelic active placebo (matching acute psychoactive/physiological cues, onset/duration, safety, and lack of therapeutic action in the target disorder), and explicitly discusses the challenge for both moderate-to-high doses and microdosing regimens [108]. In practical terms, ADHD trials could compare multiple low-dose levels (e.g., 5 vs. 10 vs. 20 µg LSD-equivalent) against a carefully chosen active placebo that mimics peripheral cues without engaging the putative therapeutic mechanism, because controlled work shows that even low doses can produce measurable effects on time perception and physiology while remaining only modestly perceptible—suggesting there may be dose ranges where masking is more feasible [74].

A second target is to measure expectancy and quantify unblinding rigorously, prospectively, and in a standardized manner rather than post hoc or informally. Psychedelic-trial methodology papers recommend standardized expectancy tools and standardized blinding metrics, because many historical trials either did not measure these constructs or did so unsystematically [47,81]. On the expectancy side, validated instruments already exist and can be applied directly to psychedelic ADHD trials: the Stanford Expectations of Treatment Scale (SETS) measures positive and negative outcome expectancy and has predictive validity in clinical-trial contexts [109]. The newer Treatment Expectation Questionnaire (TEX-Q) provides a multidimensional assessment of expectations (benefit, positive impact, adverse events, negative impact, process, behavioral control), which is particularly suitable for interventions where fear of adverse experiences and beliefs about “personal responsibility for success” may shape outcomes [110]. On the blinding side, the Bang Blinding Index offers an arm-specific, interpretable measure of blinding success that can detect differential unblinding across arms—an especially relevant feature for psychedelics [111]. Importantly, these measures should be repeated over time (e.g., after dose 1, mid-treatment, end-treatment, and follow-up), as unblinding often progresses as participants learn the intervention’s cue profile.

A third target is to move beyond symptom checklists as the sole “truth source” by improving ecological validity and reducing the sensitivity of endpoints to transient appraisal shifts. This does not mean symptom scales should be abandoned—regulators and clinicians need them—but rather that ADHD psychedelic trials should incorporate objective and near-real-time functional measures that are harder to move via belief alone. Smartphone-based ecological momentary assessment (EMA) and mobile sensing are increasingly used to capture symptom dynamics in everyday life, with protocols already registered specifically for adult ADHD that combine EMA with activity tracking. Broader EMA methodology shows that frequent sampling reduces recall bias. It can quantify within-day variability and context sensitivity—features that are central to ADHD and likely to be sensitive to any actual treatment effect [112,113]. Complementary approaches, such as app-validated symptom instruments for long-term monitoring, or immersive tasks (e.g., VR paradigms), are being developed explicitly to address the limited ecological validity of standard neuropsychological tests in adult ADHD [114,115]. For psychedelic microdosing trials—where effects may be subtle and context-linked—these “in-the-wild” measures are not just nice-to-have; they may be essential to detect reliable changes in initiation, persistence, time-on-task, sleep regularity, and real-world distractibility.

A fourth target is trial architecture that matches the intervention’s likely effect profile. If microdosing effects are expected to be small-to-moderate and heterogeneous, conventional parallel-group designs may be underpowered unless sample sizes are large and measurement reliability is high. The microdosing literature has already shown that placebo-controlled designs can be implemented outside laboratory settings (e.g., self-blinding approaches), offering a scalable path to larger samples, while still permitting randomized inference. Hybrid strategies are plausible: initial small mechanistic studies can optimize dose/schedule and identify biomarkers or proximal behavioral signals. Larger pragmatic randomized trials can test effectiveness in clinically representative ADHD populations. Methodological recommendations for psychedelic trials explicitly encourage improving rigor via dose–response logic, active comparators, and designs that address expectancy rather than assuming it away [47]. In ADHD specifically, where placebo response is strong and variable, enrichment strategies (e.g., baseline stability periods, repeated baseline sampling, or randomized run-in designs) can help distinguish regression-to-the-mean from treatment-linked change.

A fifth target is mechanistic and mediator-focused modeling that is explicitly transdiagnostic. If the most plausible pathway to symptom improvement is through changes in emotion regulation, stress reactivity, acceptance, and behavioral flexibility, then trials should measure these processes repeatedly and formally test mediation rather than treating them as secondary outcomes. This aligns with broader psychedelic clinical-trial reform arguments, which emphasize that expectancy and context shape outcomes and should therefore be modeled—not merely controlled. For microdosing specifically, the field needs clearer pharmacodynamic anchoring: acute microdose studies show dose-dependent physiological and perceptual effects, but repeated-dose studies often show little durable change, suggesting that “how much” and “how often” may matter more than assumed, and that outcomes may depend on whether doses are perceptible enough to induce learning/behavior change (which also increases expectancy confounding). This tension is exactly why dose-finding, schedule optimization, and mechanistic endpoints (sleep, arousal, cognitive control under distraction, reward sensitivity) are high-value in early-phase ADHD studies.

A sixth target is interaction-aware recruitment and stratification, because ADHD is heterogeneous and often comorbid, and many prospective participants will be taking stimulants, atomoxetine, antidepressants, or other agents. Rather than excluding these realities entirely (which harms generalizability), future trials can stratify by medication class or stability and predefine subgroup analyses, while building conservative safety monitoring into protocols. Alongside this, the field must normalize high-quality harm reporting. Updated CONSORT Harms guidance stresses transparent methods of adverse-event ascertainment and complete reporting, which is particularly important for psychedelics, given the gap between controlled-trial safety impressions and the messy realities of community use [116]. More generally, a recent review shows that psychedelic RCTs have historically been inconsistent in reporting on blinding and expectancy [47]; applying modern reporting standards to both outcomes and harms would materially improve interpretability.

Future ADHD-focused psychedelic research should move beyond diagnostically broad samples and predefine stratification targets that are mechanistically and clinically interpretable. At minimum, trials should distinguish: (1) predominantly inattentive versus hyperactive-impulsive/combined presentations; (2) participants with versus without prominent emotional dysregulation; (3) participants with versus without clinically relevant sleep/circadian disturbance; and (4) participants with versus without substance-use comorbidity. These are not merely descriptive subgroupings. They index partially separable pathways of impairment and may determine whether an observed change reflects improvement in core ADHD symptoms, affective dysregulation, sleep-related functioning, or addiction-related processes. Without such stratification, true signals may be diluted in heterogeneous samples and apparent benefits may be misattributed to ADHD symptom improvement when they primarily involve adjacent transdiagnostic domains.

Accordingly, future trials should prespecify moderator and mediator analyses for these strata, rather than treating heterogeneity as post hoc noise. A translationally stronger design would pair symptom outcomes with mechanistically matched measures: ecological attention/task-persistence outcomes for core ADHD symptoms, validated emotion-dysregulation measures for affective liability, sleep diaries/actigraphy/circadian markers for sleep-related pathways, and substance-use outcomes where relevant. Without such matching of outcomes to hypothesized pathways, true signals may be diluted in phenotypically mixed samples, making it difficult to determine whether observed changes reflect core ADHD improvement or adjacent transdiagnostic domains. 

A specific future-research priority is sleep and circadian regulation. This pathway is especially relevant in ADHD because sleep problems are common, are associated with greater symptom severity and poorer quality of life, and may interact bidirectionally with emotion regulation and attentional control [117,118]. Moreover, work targeting circadian misalignment in ADHD suggests that improving sleep timing can reduce subjective ADHD symptoms, indicating that symptom ratings may be responsive to downstream physiological regulation even when core neurocognitive liabilities are not directly altered [119]. For psychedelic studies, this means sleep should not be treated as a peripheral side variable. Instead, future trials should assess sleep prospectively using validated questionnaires, sleep diaries, actigraphy, and, where feasible, circadian phase markers, both to detect potential benefit and to test whether any symptom changes are mediated by improved sleep regularity, arousal regulation, or daily routine stabilization. In other words, sleep may represent an important mechanistic and functional target for future ADHD-focused psychedelic research, even though it remains insufficiently addressed in the current evidence base.

A further design question is whether future ADHD-focused research should prioritize repeated low-dose regimens or higher-dose psychedelic models. Based on the current evidence, low-dose approaches should probably be the first priority for controlled ADHD trials, not because they have already shown convincing efficacy, but because they are better aligned with the population and the existing signal being investigated. Most ADHD-focused studies to date have examined low-dose LSD or microdosing-like use rather than full-dose psychedelic treatment, and the central unresolved question is therefore whether any incremental pharmacological benefit can be demonstrated once expectancy, self-selection, and measurement artifacts are adequately controlled [33,34,35,36]. Low-dose designs are also more straightforward to standardize, more compatible with repeated measurement of attention and day-to-day functioning, and more feasible for testing in adults with ADHD who may have comorbidity, polypharmacy, occupational demands, and limited suitability for prolonged supervised high-intensity sessions. At the same time, they pose a major inferential challenge because subtle but still perceptible acute effects may preserve expectancy while producing only modest or transient pharmacological signals, making masking particularly difficult.

Higher-dose approaches should not be dismissed; rather, they should be treated as a distinct therapeutic model rather than as a simple extension of microdosing. The retreat-based ayahuasca study suggests that non-microdosing psychedelic exposure may also be associated with improvement in self-reported ADHD-like symptoms. Still, its design does not allow one to determine whether any change reflected pharmacology, ceremonial context, expectancy, supportive ritual structure, acute transformative experience, or their interaction [37]. More broadly, high-dose models may prove more effective than low-dose ones if clinically meaningful benefit depends on pronounced acute subjective effects, shifts in self-processing, or intensive psychotherapeutic integration—mechanisms that may not be engaged by repeated sub-perceptual dosing. However, these same features also make high-dose studies harder to blind, harder to standardize, and more difficult to interpret in ADHD populations with medication use, psychiatric comorbidity, and variable functional impairment. For that reason, the most informative next step is probably not to choose one model prematurely, but to study them sequentially and explicitly as different interventions: first, rigorous placebo-controlled dose-finding studies of low-dose regimens; second, if justified, carefully screened and highly standardized trials of full-dose psychedelic treatment with defined psychotherapeutic support, clear acute and cumulative endpoints, and direct comparison of pharmacological versus contextual contributions.

## 5. Mechanisms of Action of Psychedelics in ADHD

### 5.1. Neurotransmitter Regulation

LSD, psilocybin, and ayahuasca (via its active component DMT) are classical serotonergic psychedelics whose primary pharmacological action is agonism of the serotonin 5-HT_2_A receptor [120,121]. This receptor is densely expressed in the prefrontal cortex (PFC) [122,123], a region crucial for executive function and attention regulation [124]. By stimulating 5-HT_2_A receptors on PFC pyramidal neurons, psychedelics can modulate frontal cortex activity and downstream neurotransmitter release. Notably, 5-HT_2_A activation in PFC can enhance dopaminergic transmission: it has been shown to excite midbrain dopamine neurons and increase dopamine release in reward pathways [125]. This is of particular interest in ADHD, a condition marked by dopamine dysregulation [1], since increasing frontal dopamine is a convergent mechanism of conventional stimulants [126]. Indeed, LSD uniquely binds not only to serotonergic sites but also to dopamine receptors (e.g., D1/D2 receptors) [127,128]. LSD’s mixed receptor profile may produce mild psychostimulant effects alongside its serotonergic activity. For example, low doses of LSD (10–20 µg) in healthy adults elicited subjective increases in energy, “amphetamine-like” feelings, and greater “intellectual efficiency” on psychometric scales [129,130]. Consistent with these effects, many microdosing users report enhanced concentration and productivity [131]. Psilocybin (converted in vivo to psilocin) has a more selective serotonergic action (high affinity for 5-HT_2_A/2C/1A receptors) and minimal direct dopamine receptor binding. Still, it too may indirectly influence dopamine pathways via 5-HT_2_A-mediated network effects [132]. Ayahuasca, a brew containing DMT and MAO inhibitors (harmala alkaloids), likewise engages the 5-HT system. Its DMT component acts on 5-HT_2_A/1A receptors similarly to psilocin, while MAO-A inhibition elevates synaptic monoamines broadly. This combined mechanism could acutely boost serotonin and possibly dopamine/norepinephrine levels, complementing the direct 5-HT_2_A stimulation [133,134]. At the same time, these pharmacological observations should be interpreted conservatively in relation to ADHD. Although serotonergic psychedelics can influence catecholaminergic signaling and prefrontal network dynamics, this does not mean that ADHD should be understood as a serotonergic disorder or that serotonergic modulation is, by itself, a validated treatment pathway for ADHD. The translational gap here is substantial: receptor-level and systems-level plausibility does not establish disorder-specific clinical efficacy, particularly given that the current ADHD literature contains only one randomized placebo-controlled trial, which did not demonstrate superiority over placebo. For this reason, the mechanistic account presented in this review is best regarded as a framework for generating testable hypotheses about possible pathways of action, not as evidence that these mechanisms have been shown to produce clinically meaningful benefit in ADHD. In sum, through serotonergic agonism (common to LSD, psilocybin, DMT) and ancillary dopaminergic/monoaminergic effects (notably with LSD and ayahuasca), these psychedelics engage molecular pathways that overlap with those implicated in ADHD. The heightened serotonin signaling is hypothesized to alleviate core symptoms—one pilot study speculated that DMT’s action on 5-HT receptors was key to the reductions in inattention and hyperactivity seen after ayahuasca sessions [37]. Psychedelics, by concurrently modulating dopamine (especially with LSD), may further align with the dopaminergic mechanism of traditional ADHD medications. This unique polypharmacology provides a neurochemical rationale for their therapeutic exploration in ADHD.

### 5.2. Brain Network Modulation and Connectivity

Beyond receptors, psychedelics induce distinctive changes in large-scale brain networks that contrast with the baseline patterns seen in ADHD. A hallmark is disruption of the default mode network (DMN)—the interconnected set of midline regions (posterior cingulate cortex, medial prefrontal cortex, etc.) active during mind-wandering and internally directed thought [135,136,137]. Excessive or dysregulated DMN activity has been linked to ADHD, contributing to lapses in attention and an inability to appropriately suppress internal distractions [138,139]. Acute psychedelic states markedly diminish DMN functional connectivity. Psilocybin and LSD both decrease coherent activity within the DMN (particularly decoupling the mPFC and PCC hubs) while increasing integration between normally segregated networks [140,141,142]. Under LSD, for instance, the medial–posterior core of the DMN shows significantly reduced within-network coupling, accompanied by a surge in global connectivity across brain networks [143]. This translates to a state o enhanced communication between the DMN and task-positive networks that are usually anti-correlated. From a cognitive standpoint, such DMN disintegration and network “reset” may help diminish the internally focused, stimulus-independent mentation that often competes with external attention in ADHD. Simultaneously, psychedelics can upregulate attention and control networks. Neuroimaging has shown that LSD (75 µg) increases the dynamical complexity and fractal dimension of activity within the dorsal attention network and frontoparietal (executive control) network, essentially energizing these task-focused circuits [144]. This is noteworthy because ADHD is associated with hypoactivation of frontal–parietal control networks during cognitive tasks and hyperactivity of the DMN at rest; psychedelics acutely push the brain toward the opposite pattern (higher engagement of attention networks, reduced self-referential network dominance).

Importantly, some network effects persist beyond the acute drug window and may underlie longer-term benefits. Ayahuasca, for example, not only transiently disrupts normal DMN function during the psychedelic experience [145], but repeated use has been associated with structural and connectivity changes in DMN nodes. Long-term ayahuasca users exhibit increased cortical thickness in the anterior cingulate cortex (a region involved in attentional control) and a relative thinning of the PCC [146]. A thinner PCC (core of the DMN) and a thicker ACC could reflect a functional re-balancing of network dynamics in favor of improved attention and reduced self-focus. Indeed, ayahuasca has been reported to increase local connectivity and entropy while decreasing global network integration, suggesting a more flexible, less rigidly segregated brain state [147]. Such network-level reorganization aligns with the theory that psychedelics relax high-level default network activity (“resetting” overactive prediction networks) and facilitate greater bottom-up information flow (REBUS model) [148].

### 5.3. Neuroplasticity and Synaptic Adaptations

One of the most intriguing mechanistic aspects of psychedelics is their ability to stimulate neuroplasticity, which may lead to enduring neural adaptations relevant to ADHD. A growing body of preclinical research shows that classical psychedelics are potent “psychoplastogens,” meaning they induce rapid and lasting changes in neuronal structure and gene expression [149,150,151]. In cellular and animal models, LSD, psilocybin, and DMT all promote the expression of plasticity-related genes, such as BDNF (brain-derived neurotrophic factor) and immediate-early genes (e.g., EGR-1), within hours of exposure [152,153,154]. This triggers molecular cascades (e.g., the TrkB receptor, and mTOR signaling) that lead to robust synaptogenesis and dendritic growth [155,156]. Experiments have demonstrated that these psychedelics can cause neurons, especially in the prefrontal cortex, to sprout new dendritic spines and form new synaptic connections, in a manner comparable to or exceeding pro-plasticity drugs like ketamine [157,158,159]. They can also enhance synaptic strength, as evidenced by facilitation of long-term potentiation in rodent brain slices [160,161]. At the level of behavioral level, these changes correlate with improved learning and cognitive flexibility in animal models. For instance, repeated DMT administration in mice upregulates early-growth response genes linked to attention processes and has been associated with improved performance on memory tasks in some studies, implying functional circuit upgrades [162]. These molecular and structural neuroplastic changes are highly relevant to ADHD, which has been conceptualized in part as a disorder of delayed neurodevelopment and cortical maturational lag [163]. By accelerating neuronal growth and network reorganization, psychedelics might help “update” neural circuits that underlie attention and impulse control.

There is also evidence of psychedelic-induced plasticity in humans. Ayahuasca administration acutely elevates circulating BDNF levels in both healthy volunteers and depressed patients, suggesting that the brew triggers a neurotrophic response in the brain [164]. Although not all studies agree on peripheral BDNF changes, this finding supports the idea that psychedelics enter a transient plasticity-promoting physiological state. More strikingly, structural MRI studies of long-term ayahuasca users (as noted above) revealed anatomical differences in cortical thickness in frontal and posterior regions, consistent with lasting plastic changes from repeated use. These changes (increased ACC thickness, decreased PCC) mirror the functional network effects and may reflect synaptic remodeling driven by sustained 5-HT_2_A activation and growth factor upregulation. Likewise, a recent clinical trial on LSD microdosing collected evidence (through fMRI and cognitive testing) hinting that even sub-hallucinogenic doses of LSD might induce subtle neuroadaptive changes over several weeks. However, more research is needed [143].

## 6. Conclusions

The current evidence based on classical psychedelics as a treatment for adult ADHD remains small, heterogeneous, and methodologically fragile. Across five included prospective/experimental studies, the most consistent pattern is short-term improvement in self-reported ADHD symptoms and related subjective functioning in uncontrolled contexts—self-initiated microdosing cohorts and a retreat-based ayahuasca pre-postostandpost-design. However, the best-controlled evidence—a double-blind, placebo-controlled phase 2A trial of repeated low-dose LSD—showed substantial improvement in both LSD and placebo groups without a clear incremental benefit of LSD on core ADHD symptom endpoints.

A coherent interpretation is that the apparent “signal” in naturalistic settings likely reflects a mixture of influences: pharmacology may contribute for some individuals, but expectancy, self-selection, regression to the mean, assessment reactivity, and attrition/survivorship bias plausibly explain a substantial portion of the significant pre–post changes observed in uncontrolled designs. This interpretation is critical in ADHD, where placebo responsiveness on symptom ratings is known to be substantial, making short-horizon symptom-scale improvements intrinsically ambiguous unless expectancy and blinding integrity are explicitly measured and modeled.

This interpretive caution should also shape how the mechanistic material is read. Although serotonergic psychedelics plausibly affect systems that intersect with ADHD-relevant processes, the clinical literature reviewed here does not yet provide convincing disorder-specific evidence that these mechanisms translate into therapeutic benefit beyond placebo. Accordingly, the breadth of mechanistic discussion should not be taken to imply a corresponding strength of clinical support. At present, the mechanistic rationale is scientifically interesting but remains provisional and subordinate to the negative placebo-controlled efficacy finding.

Where secondary outcomes were assessed, the most replicable changes clustered in psychological process domains—well-being, emotion regulation (notably reduced expressive suppression and increased reappraisal), mindfulness-related capacities, and reduced negative emotionality—while objective cognitive evidence was sparse and did not show consistent improvement (e.g., null findings on a time-perception task). These patterns suggest that any clinically meaningful benefits—if they exist—may arise less from direct “stimulant-like” enhancement of attention and more from process-level shifts (stress reactivity, self-regulation style, emotional control, and coping) that can improve day-to-day functioning and reduce symptom endorsement, even if core neurocognitive liabilities are unchanged.

From a safety perspective, the most informative data come from the controlled low-dose LSD trial: serious adverse events were not prominent under screened, supervised conditions, but treatment-emergent adverse effects were more frequent with LSD than placebo, and some participants discontinued due to acute subjective effects—indicating that “microdose-like” regimens are not uniformly subperceptual or trivial in tolerability. In naturalistic microdosing cohorts and retreat settings, safety surveillance was insufficient to estimate adverse-event incidence, leaving significant uncertainty about real-world risk—particularly relevant for adults seeking ADHD relief who often have comorbidity and polypharmacy.

Overall, there is not yet adequate evidence to support psychedelics as an evidence-based treatment for ADHD. The field is nonetheless scientifically motivated: serotonergic psychedelics plausibly engage neurobiological systems relevant to ADHD (serotonergic–catecholaminergic interactions, large-scale network dynamics, and plasticity-related pathways), and the subjective/psychological changes observed map onto recognized contributors to adult ADHD impairment. However, translating this rationale into credible clinical claims will require next-generation trials explicitly designed to separate pharmacology from context and expectancy: improved masking (ideally active placebos), routine expectancy and blinding assessment, dose–response and schedule optimization, mechanistic and mediator-focused models, objective and ecologically valid functional outcomes, stratification for comorbidity and concurrent medications, longer follow-up, and high-quality harms reporting.

In conclusion, the current evidence base does not support psychedelics as an evidence-based treatment for adult ADHD. Positive findings are concentrated in naturalistic, self-selected, short-term studies that are highly vulnerable to expectancy and contextual confounding, whereas the strongest controlled evidence to date has not shown superiority of repeated low-dose LSD over placebo for core ADHD symptoms. The most reproducible changes reported outside controlled settings involve self-rated well-being and affective/self-regulatory domains, which may reflect effects on transdiagnostic or comorbid dimensions rather than core ADHD pathology. Future work should therefore prioritize rigorous masking, standardized dosing, objective/ecological outcome measures, systematic harm reporting, and phenotype-informed stratification capable of distinguishing core ADHD effects from changes in adjacent affective or contextual processes.

In conclusion, the reviewed literature supports a cautious, research-only stance: psychedelics remain an intriguing but unproven approach for ADHD. Until larger, rigorously controlled, mechanism-informed studies demonstrate reproducible drug-specific benefits with acceptable safety, clinical use should be considered premature, and any exploration should occur within ethically robust, well-monitored research frameworks.

## Figures and Tables

**Table 1:** Characteristics of included studies: design, setting, participants, comparators, and timepoints.

Timepoints	Comparator	Participants	Design & Setting	Study
Baseline (1–3 days before starting MD), 2 weeks, 4 weeks	No control group; within-person pre/post comparison over time. Secondary subgroup contrasts: microdosing alone vs. microdosing plus prescribed ADHD medication; ADHD with vs. without comorbidity	Baseline analyzed cohort n = 233; follow-up n = 66 at 2 weeks, n = 47 at 4 weeks; mean age 35.3 years; roughly balanced gender; mostly Europe-based, highly educated; included adults with formal ADHD diagnosis or clinically elevated ADHD symptoms interfering with daily life	Prospective naturalistic longitudinal online study; adults self-initiated psychedelic microdosing as self-medication; recruitment via microdosing website; fully remote repeated online assessments; data collected November 2020–July 2021	[33]
Baseline, ~2 weeks, ~4 weeks in both studies	Study 1: none (within-person change only). Study 2: microdosing-only vs. TAU conventional ADHD medication continuation	Study 1: analyzed baseline n = 233, follow-up n = 64 at 2 weeks and n = 44 at 4 weeks; adults with ADHD diagnosis or clinically elevated symptoms. Study 2: microdosing-only group n = 180 baseline, n = 50 at 2 weeks, n = 38 at 4 weeks; TAU group n = 37 baseline, n = 27 at 2 weeks, n = 28 at 4 weeks	Study 1: fully naturalistic prospective online study of self-initiated microdosing. Study 2: naturalistic prospective comparison of a microdosing-only subgroup vs. treatment-as-usual (TAU) conventional ADHD medication users; repeated online assessments	[34]
Baseline (1–3 days before starting), 2 weeks, 4 weeks; plus daily diary reports of microdosing	No control group; within-person pre/post comparison over time. Secondary subgroup analyses by concurrent ADHD medication use, comorbidity, and recent mindfulness practice	Analyzed baseline cohort n = 233; follow-up n = 66 at 2 weeks, n = 44 at 4 weeks; adults with formal ADHD diagnosis or severe ADHD symptoms; mostly Europe-based, highly educated, extensive prior psychedelic experience; common comorbidity	Prospective naturalistic online survey; adults about to begin self-initiated psychedelic microdosing; recruitment via microdosing information website; daily diary plus repeated surveys; Maastricht ethics approval; data collected November 2020–July 2021	[35]
Screening/baseline; efficacy assessed during treatment including week 2 and week 6; follow-up around week 10 (about 3–4 weeks post-treatment)	Placebo oral solution matched to LSD vehicle	Randomized n = 53 total: LSD n = 27, placebo n = 26; adults aged 18–65 with established ADHD diagnosis and current moderate-to-severe symptoms; mean age 37 years; 42% female	Multicenter, double-blind, placebo-controlled, parallel-group phase 2A RCT; outpatient sites in Basel, Switzerland and Maastricht, Netherlands; supervised twice-weekly dosing for 6 weeks	[36]
Pre-retreat/before first ceremony and post-retreat/morning after final ceremony	No control group; within-person pre/post retreat comparison	n = 49 completed symptom measures; n = 47 provided demographics; adults attending retreats, mostly not clinically diagnosed with ADHD (only 4 self-reported ADHD diagnosis); mean age 40.93 years; mixed sex and demographic background	Exploratory naturalistic repeated-measures observational study at Ayahuasca Foundation retreat/research centre, Iquitos, Peru; traditional Shipibo ceremonial retreat setting; pre/post assessment	[37]

**Table 2:** Characteristics of included studies: exposure, measures, outcomes, moderators, and limitations.

Limitations	Moderators	Outcomes (Summarized)	Primary Measures	Exposure	Study
Naturalistic, nonrandomized, no control group; self-selection and expectancy effects; heavy attrition; exposure highly heterogeneous and incompletely documented; outcomes mostly self-report; diary data missing for many; small follow-up samples, especially medicated subgroup	Concurrent ADHD medication slightly altered early symptom trajectory (smaller improvement at week 2, not week 4); comorbidity mainly associated with worse baseline status, not different response pattern; medication × time × interval effect observed on shortest time-perception interval, but based on very small subgroup	Self-reported CAARS scores and WHO-5 well-being improved over 2–4 weeks, but no consistent objective cognitive improvement was observed.	CAARS-S:SV (inattention, hyperactivity/impulsivity, ADHD index, DSM-IV total), WHO-5 well-being, online auditory time reproduction task	Self-initiated psychedelic microdosing over 4 weeks in a real-world setting; mostly psilocybin/truffles, smaller numbers using LSD, novel lysergamides, or ayahuasca; dosing not standardized	[33]
No randomization or blinding; naturalistic self-treatment; substantial attrition; baseline differences between groups in Study 2 (including sex balance, prior psychedelic exposure, and symptom levels); mostly self-report outcomes; heterogeneous exposure	In Study 1, changes were not significantly moderated by concurrent ADHD medication use or comorbidity; in Study 2, main comparison was group (microdosing-only vs. TAU)	Study 1: cognitive reappraisal increased, expressive suppression decreased by week 4, perspective-taking increased by week 4, personal distress decreased at week 2 only; empathic concern/fantasy unchanged. Study 2: microdosing group showed steeper improvement than TAU on all CAARS ADHD indices and had lower symptom scores by week 4; for emotion regulation, the most consistent advantage was lower expressive suppression in microdosing vs. TAU; empathy changes were limited and not clearly microdosing-specific	ERQ (cognitive reappraisal, expressive suppression), IRI (perspective-taking, fantasy, empathic concern, personal distress); in Study 2 also CAARS-S:SV ADHD symptom indices	Study 1: self-initiated microdosing over 4 weeks; Study 2: microdosing-only subgroup compared with stable conventional ADHD medication (TAU) over the same interval	[34]
Naturalistic, uncontrolled design; no placebo/control group; heavy attrition; self-selected sample with extensive prior psychedelic and mindfulness experience; exposure not standardized; self-report trait measures; incomplete dosing diary data	Concurrent ADHD medication associated with lower total mindfulness/non-judging at week 2 but not baseline or week 4; comorbidity did not materially alter change trajectories; recent mindfulness practice explained some apparent mindfulness gains	Total mindfulness increased by week 2 and further by week 4; after accounting for recent mindfulness practice, the most robust facet increases were describing and non-judging; neuroticism decreased by week 4; other personality traits showed little reliable change after correction	FFMQ-15 (total and five mindfulness facets), BFI-10 (Big Five personality traits)	Self-initiated microdosing over 4 weeks; mostly psilocybin/truffles, smaller numbers LSD/novel lysergamides/ayahuasca; flexible real-world dosing	[35]
Small sample; possible strong expectancy/placebo effects; blinding only partially successful; fixed 20 μg dose may have been above truly “subperceptual” range for some; short duration; mostly one site contributed participants	Sensitivity analyses explored baseline severity; expectancy/blinding was important: many participants guessed LSD, and believing one had received LSD was associated with nominally larger symptom reductions	Both LSD and placebo groups improved over time, but LSD did not outperform placebo on the primary endpoint or secondary ADHD symptom measures; improvements persisted to follow-up in both groups; LSD produced stronger acute subjective effects.	Primary: AISRS clinician-rated ADHD symptoms; secondary: CGI-S, CAARS, ASRS; safety/adverse events; acute subjective effect measures	20 μg LSD vs. placebo, orally, twice weekly for 6 weeks (12 supervised doses total), double-blind, parallel-group outpatient trial	[36]
Mostly not a clinically diagnosed ADHD sample; only 4 participants reported ADHD; no control group; self-report only; retreat includes many nonspecific influences (setting, expectation, diet, travel, group effects); variable dosing and ceremonies; medication discontinuation/absence during retreat complicates interpretation	No significant moderation by retreat length or number of ceremonies; sex-stratified analyses suggested possible descriptive differences in which symptom domains changed more, but findings were exploratory	Significant pre–post reductions in inattention, hyperactivity/impulsivity, and total ADHD-like symptoms in the full sample, with medium-to-large effect sizes; reductions also seen separately in men and women; descriptive improvement in 3 of 4 participants with self-reported ADHD	ASRS inattention, hyperactivity/impulsivity, total symptoms, pre-retreat vs. morning after final ceremony	Participation in a traditional ayahuasca retreat in Peru; number of ceremonies and retreat length varied; brew not chemically standardized; standard ADHD medication absent during retreat	[37]

**Table 3:** ROBINS-I risk of bias.

Study	Bias Due to Confounding	Bias in Selection of Participants into the Study	Bias in Classification of Interventions (Exposure)	Bias Due to Deviations from Intended Interventions	Bias Due to Missing Data	Bias in Measurement of Outcomes	Bias in Selection of the Reported Result
[33]	Critical	Serious	Serious	Moderate to Serious	Critical	Serious	Moderate
[34] study 1	Critical	Serious	Serious	Moderate to Serious	Critical	Serious	Moderate
[34] study 2	Serious	Serious	Moderate to Serious	Moderate	Serious to Critical	Serious	Moderate
[35]	Critical	Serious	Serious	Moderate to Serious	Critical	Serious	Moderate
[37]	Critical	Serious	Moderate to Serious	Serious	Low to Moderate (unclear)	Serious	Moderate to Serious (unclear)

**Table 4:** RoB-2 risk of bias.

Study	Bias Arising from the Randomization Process	Bias Due to Deviations from Intended Interventions	Bias Due to Missing Outcome Data	Bias in Measurement of the Outcome	Bias in Selection of the Reported Result
[36]	Low	Some concerns	Low	Some concerns	Some concerns

## Data Availability

No new data were created or analyzed in this study. Data sharing does not apply to this article.
